# TIE-UP-SIN: a novel method for enhanced identification of protein–protein interactions

**DOI:** 10.3389/fmicb.2025.1657647

**Published:** 2025-09-01

**Authors:** Maximilian Schedlowski, Stephan Michalik, Tilly Hoffmüller, Marco Harms, Leif Steil, Kristin Surmann, Christian Hentschker, Manuela Gesell Salazar, Uwe Völker, Alexander Reder

**Affiliations:** Department Functional Genomics, Center for Functional Genomics of Microbes, Interfaculty Institute for Genetics and Functional Genomics, University Medicine Greifswald, Greifswald, Germany

**Keywords:** protein–protein interactions, heavy nitrogen metabolic labeling, formaldehyde crosslinking, *in vivo* crosslinking, mass spectrometry, affinity purification-mass spectrometry

## Abstract

Proteins function through complex interaction networks that govern nearly all aspects of cellular physiology. Identifying protein–protein interactions (PPIs) under native conditions remains challenging due to the transient nature of many complexes and technical limitations of conventional approaches. We present TIE-UP-SIN (Targeted Interactome Experiment for Unknown Proteins by Stable Isotope Normalization), a robust and reproducible method for *in vivo* identification of PPIs. This approach combines metabolic labeling with ^15^N isotopes, reversible *in vivo* formaldehyde crosslinking, affinity purification, and quantitative mass spectrometry. TIE-UP-SIN is specifically designed to preserve transient or weak interactions during purification and to quantify interaction partners using internal light/heavy peptide ratios, reducing experimental variability and increasing reproducibility across biological replicates. The method employs a triple-sample design (WT/WT, Bait/WT, Bait/Bait) to distinguish specific from non-specific interactors. Peptide-level L/H ratios are normalized against sample-specific factors, aggregated at the protein level, and statistically analyzed using moderated testing. This strategy enables reliable detection of differential PPIs across physiological states, even in organisms with limited labeling options. We demonstrate the utility of TIE-UP-SIN by mapping interaction partners of the essential housekeeping sigma factor RpoD (SigA) under control and ethanol stress conditions. Known partners such as RNA polymerase subunits (RpoA, RpoB, RpoC) were robustly enriched, while potential novel candidates, including ClpX and AcpA, were detected at lower abundance. TIE-UP-SIN offers a simple, cost-effective, and modular platform for quantitative interactome analysis and can be adapted to a wide range of bacterial and non-bacterial systems. Compared to established approaches such as label-free IP–MS or proximity-based labeling methods, TIE-UP-SIN is intended as a complementary option. Its combination of specific control, robust quantification, and suitability for low-input material provides an additional tool within the broader proteomics workflow collection.

## 1 Introduction

Proteins are essential to cellular processes, often functioning through protein–protein interactions (PPIs), which form intricate networks and highly sophisticated heterogeneous protein structures that regulate various biological activities. Prior to the advent of proteomics, identifying PPIs was a laborious and time-consuming task. Classical methods, such as X-ray crystallography and NMR spectroscopy, have long provided high-resolution spatial and structural information about protein complexes, while surface plasmon resonance (SPR) (Liedberg et al., 1983), biolayer interferometry (BLI) (Abdiche et al., 2009), isothermal titration calorimetry (ITC) (Wiseman et al., 1989), and Förster resonance energy transfer (FRET) (Stryer and Haugland, 1967) have enabled the quantification of binding affinities and kinetics. These methods are constrained by their low throughput and reliance on prior knowledge of interaction partners, rendering them unsuitable for scaling up to comprehensive proteome studies. To address these limitations, high-throughput techniques such as protein microarrays (MacBeath and Schreiber, 2000), phage display (Smith, 1985), and the two-hybrid screening systems (Fields et al., 1989) were developed. Although these methods are easier to handle and are broadly applicable, they rely on *in vitro* systems or heterologous expression systems, and the physiological relevance of their findings can be limited by factors such as the absence of cellular context, lack of post-translational modifications, and artificial experimental conditions. Therefore, findings from these systems are often complemented with additional *in vivo* studies to validate and fully understand the biological significance of the interactions, leading to time-consuming downstream analyses. In addition, advanced *in silico* methods such as AlphaFold have recently emerged as powerful tools for investigating protein–protein interactions (Senior et al., 2020; Jumper et al., 2021; Abramson et al., 2024). These computational approaches can predict the structure of protein complexes with remarkable accuracy, thereby, offering valuable insights into the interaction interfaces and dynamics without the need for extensive experimental setup. But these *in silico* models must still be combined with high-throughput experimental approaches to verify and test protein–protein interactions under true *in vivo* conditions.

Over the past decades, mass spectrometry (MS) has undergone significant advancements, which revolutionized proteomics and enabled high-throughput identification and quantification of PPIs. These advances in mass spectrometry (MS) technologies, including data-independent acquisition (DIA) and tandem MS/MS, together with the concurrent technical improvements in MS instrumentation, have substantially enhanced the sensitivity, accuracy, and throughput of protein–protein interaction (PPI) detection. These innovations allow for the comprehensive analysis of complex protein networks, detecting a wider range of interactions, from abundant proteins to low-abundance or transient interactions that were previously undetectable. Additionally, advancements in quantitative proteomics - including isotope labeling and label-free quantification - have provided workflows to measure interaction dynamics *in vivo*, offering critical insights into the functional relevance of PPIs under physiological conditions.

In recent years, techniques like affinity purification coupled with mass spectrometry (AP-MS) (Gavin et al., 2002; Gingras et al., 2007) and cross-linking mass spectrometry (XL-MS) (Sinz, 2003) have emerged as powerful tools for identifying PPIs. AP-MS excels at isolating specific protein complexes from cells and identifying interaction partners in near-native conditions. However, this method primarily captures stable interactions, while transient or weakly interacting partners are potentially neglected. Formaldehyde-mediated affinity-purification mass spectrometry (FM-AP-MS), in which formaldehyde stabilizes labile assemblies so that intact complexes can be captured on an affinity matrix prior to LC–MS/MS also allows for the identification of transient interactions. In contrast, XL-MS utilizes chemical cross-linkers to covalently thether residue pairs in proximal protein regions and locates the resulting cross-linked peptides with dedicated search engines such as XiSearch/XiView. XL-MS delivers residue-level distance restraints, whereas AP-MS or FM-AP-MS recover intact protein–protein-interactions without necessarily identifying inter-peptide cross-links. An influential precursor to our workflow is the Strep–protein interaction experiment (SPINE), which couples reversible *in vivo* formaldehyde cross-linking with Strep-tag affinity purification to stabilize and recover native complexes for MS identification with low background. Originally demonstrated in *Bacillus subtilis*, SPINE established that short FA cross-linking plus highly specific Strep/Strep-Tactin capture can provide a clean snapshot of *in vivo* PPIs (Herzberg et al., 2007).

FM-AP-MS and AP-MS have both been independently integrated with stable isotope labeling by amino acids in cell culture (SILAC), giving rise to methods like quantitative AP-MS (qAP-MS). A combination of FM-AP-MS and heavy isotope metabolic labeling would not only capture a broader spectrum of PPIs, including both stable and transient interaction, in their native cellular environments. But would also simplify normalization and statistics due to providing heavy/light ratios instead of intensities. Moreover, this integration would enhance the robustness of interaction data by confirming physical proximity through cross-linking, adding an additional layer of confidence to the identified PPIs.

In addition to AP-MS and XL-MS, proximity-dependent labeling techniques such as BioID (Roux et al., 2012) and APEX (Rhee et al., 2013) have become widely used to produce proximal protein data by quantitative biotinylproteomics in living cells. These methods rely on the enzymatic biotinylation of proximal proteins and can offer excellent spatial resolution. However, they require heterologous expression of fusion constructs and often result in background biotinylation over extended labeling periods. Furthermore, BioID and APEX provide limited information on interaction strength or stoichiometry and are not inherently quantitative.

To address these limitations, we developed TIE-UP-SIN (Targeted Interactome Experiment for Unknown Proteins by Stable Isotope Normalization), a FM-AP-MS approach integrating stable isotope metabolic labeling, reversible formaldehyde crosslinking (Sutherland et al., 2008), affinity purification, and high-resolution mass spectrometry. TIE-UP-SIN preserves native expression levels and captures weak or transient interactions *in vivo*, while isotope-based quantification enables precise and reproducible measurements. By normalizing light-to-heavy (L/H) peptide ratios within the same sample, TIE-UP-SIN minimizes run-to-run variability and batch effects common to label-free approaches. The method allows for reliable discrimination between specific and nonspecific interactors through stringent experimental controls and statistical filtering, supporting confident identification of relevant PPIs under different, physiologically relevant conditions.

Uniform heavy-nitrogen (^15^N) metabolic labeling allows the light and heavy cultures to be mixed prior to affinity purification, embedding a 1∶1 internal reference that carries through every wash and cross-link-reversal step. This mix-before-pull-down design produces ratio-based quantification within a single chromatogram, reducing inter-run variability and instrument time relative to *in vitro* peptide-level tags such as TMT or dimethyl.

**Figure 1:**
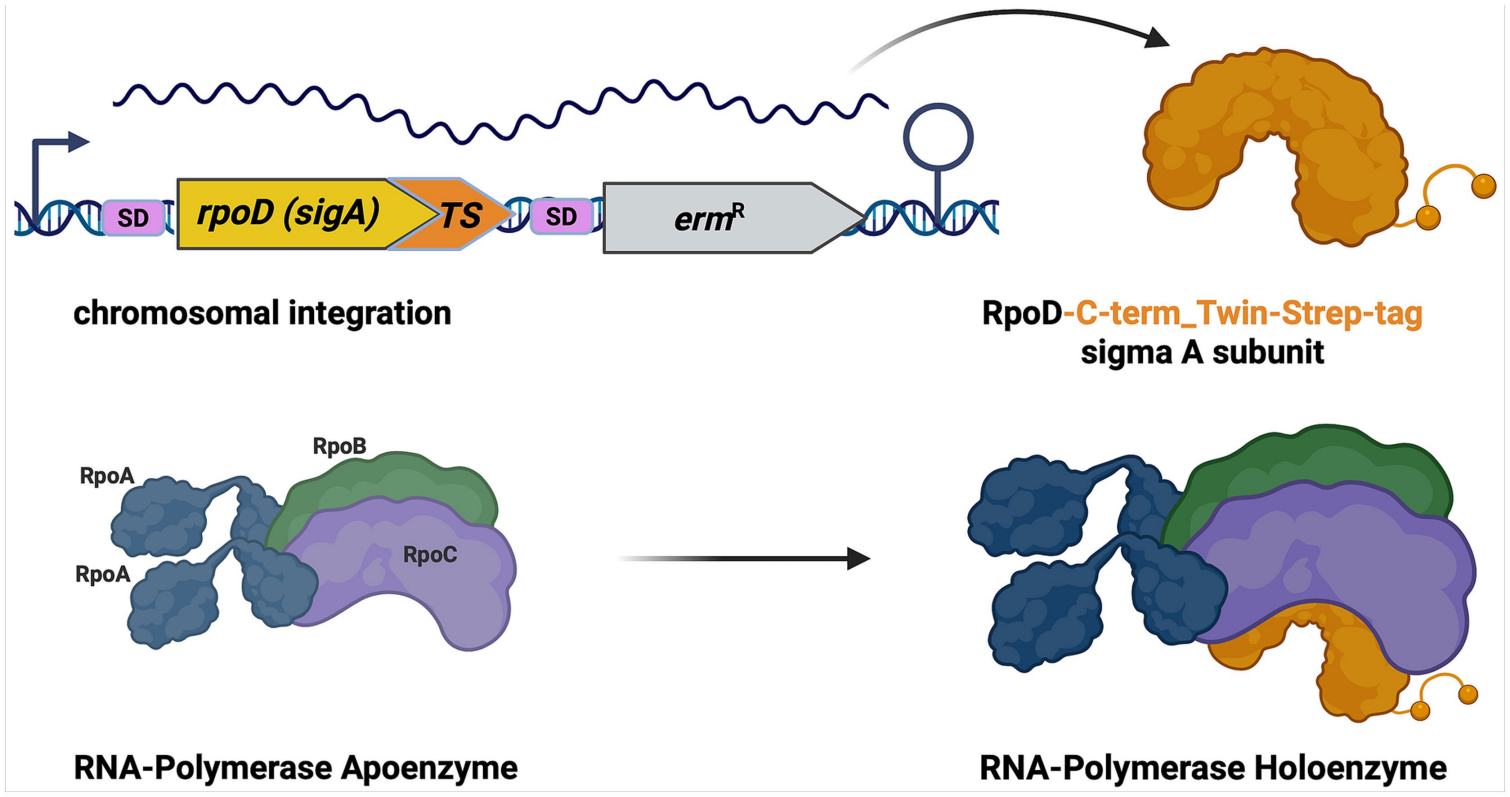
Schematic representation of C-terminal Twin-Strep-tagged SigA expression and interaction with RNA polymerase. The figure illustrates the genetic integration of the C-terminal Twin-Strep-tag (TS) into the *rpoD* (*sigA*) gene. Upon expression, the SigA protein is tagged with the Twin-Strep sequence, allowing for efficient affinity purification. At the protein level, the Twin-Strep-tagged SigA is supposed to form a complex with the RNA Polymerase Apoenzyme. The interaction is visualized, showing the positioning of the modified SigA factor within the RNA Polymerase Holoenzyme.

To demonstrate the effectiveness of TIE-UP-SIN, we applied it to investigate primary interaction partners of the essential house-keeping sigma factor SigA from *Bacillus subtilis*, which was chromosomally tagged with a C-terminal Twin-Strep-tag (TS) (Schmidt et al., 2013). Known primary interaction partners of SigA (Haldenwang, 1995; Collins et al., 2023) include RpoB, RpoC, and RpoA - the core subunits of the RNA polymerase holoenzyme (Bae et al., 2015; Johnston et al., 2009) (Figure 1). Our *in vivo* TIE-UP-SIN analysis of SigA interactions, conducted under two distinct crosslinking conditions and two physiological states - exponential growth as well as harsh physical stress caused by addition of ethanol - demonstrate that the results of our streamlined and efficient PPI assay align with established findings and show interesting clues to new interactions.

## 2 Materials and methods

### 2.1 Bacterial strains and genetical modifications

Starting with the *B. subtilis* wildtype strain BSB (Nicolas et al., 2012), the bait strain SigA-TS was constructed by chromosomal integration of a DNA fragment including the native *sigA* sequence with a translational fusion of a C-terminal Twin-Strep-tag sequence (WSHPQFEKGGGSGGGSGGSAWSHPQFEK) together with a downstream transcriptional fusion of an erythromycin resistance cassette flanked by 800 bp sequences complementary to the genomic region up- and downstream of *sigA,* allowing integration by a double cross-over event. A detailed description of the DNA sequences and primers used for the integration can be found in the Supplementary Table S1 SigA-Construction. All oligonucleotides were synthesized and purchased from Biolegio (Nijmegen, Netherlands).

### 2.2 Cultivation of bacteria, metabolic labeling, crosslinking, and protein extraction

To ensure as complete as possible metabolic labeling, bacteria were cultured in either BioExpress® Bacterial Cell Medium (unlabeled) (Cambridge Isotope Laboratories, Inc.) or labeled BioExpress® Bacterial Cell Medium (U-^15^N, 98%) (10X concentrate) (Cambridge Isotope Laboratories, Inc.), starting from an overnight serial dilution culture. A pre-culture was inoculated to generate sufficient volume for the main cultures. At an optical density at 540 nm (OD_540 nm_) of 0.8, two samples of 8 OD units each (calculated as OD_units_/OD_measured_ = V_harvest_) were harvested from every culture. The crosslinking FA solution was freshly prepared with Paraformaldehyde, extra pure (Carl Roth GmbH + Co. KG) as 4% (w/v) in 50 mM HEPES Bioscience-Grade (Carl Roth GmbH + Co. KG), and the pH was adjusted to pH 8. The harvested culture was directly transferred into a 50 mL reaction tube containing the appropriate amount of the 4% (w/v) FA solution for a final FA concentration of 0.2% (w/v) or 0.4% (w/v) (Grajales et al., 2015). The tube was inverted several times and incubated at 37°C for 15 min, followed by centrifugation at 6462 x g for 3 min. There was no quenching used to prevent over-cross-linking. The supernatant was discarded, the pellet was shock-frozen in liquid nitrogen (LN_2_), and stored at −80°C. To induce ethanol (EtOH) stress, bacterial cultures were treated with 4% (v/v) EtOH (final concentration) at an OD_540 nm_ of 0.8, followed by cell harvest 10 min later, while all other culture conditions remained identical to the unstressed control.

Immediately before cell disruption, the appropriate ^14^N-labeled and ^15^N-labeled pellets were combined as described in Table 1. Therefore, a frozen pellet (8 OD units) was resuspended in 100 μL disruption buffer (20 mM HEPES pH 8) and then used to resuspend the second frozen pellet it was to be combined with. The combined, resuspended pellets were then transferred into a 4.8 mL Teflon Vessel, precooled and filled with LN_2_, containing an 8 mm steel ball. The cells were mechanically disrupted using a Dismembrator MM400 (Retsch GmbH) at 2600 rpm for 3 min.

**Table 1:** Mixing table for the differently labeled cell pellets (^14^N and ^15^N).

Cell pellet mixing table			
Replicate	Sample	Pellet ^14^N (light)	Pellet ^15^N (heavy)
1	WT/WT control	WT ^14^N BR1	WT ^15^N BR1
	SigA/WT experiment	SigA-TS ^14^N BR1	WT ^15^N BR1
	SigA/SigA control	SigA-TS ^14^N BR1	SigA-TS ^15^N BR1
2	WT/WT control	WT ^14^N BR2	WT ^15^N BR2
	SigA/WT experiment	SigA-TS ^14^N BR2	WT ^15^N BR2
	SigA/SigA control	SigA-TS ^14^N BR2	SigA-TS ^15^N BR2
3	WT/WT control	WT ^14^N BR3	WT ^15^N BR3
	SigA/WT experiment	SigA-TS ^14^N BR3	WT ^15^N BR3
	SigA/SigA control	SigA-TS ^14^N BR3	SigA-TS ^15^N BR3
4	WT/WT control	WT ^14^N BR4	WT ^15^N BR4
	SigA/WT experiment	SigA-TS ^14^N BR4	WT ^15^N BR4
	SigA/SigA control	SigA-TS ^14^N BR4	SigA-TS ^15^N BR4

Each pellet has roughly the same cell amount according to harvesting 8 OD-units. BR is short for biological replicate.

Across the four biological replicates the SigA-tag strain was grown in ^15^N medium and the control strain in ^14^N medium; a reciprocal (label-swapped) layout was not pursued because initital lysate mixes showed no isotope-specific detection bias and the mix-before-affinity-purification strategy exposes both isotopologues to identical purification and cross-link reversal steps.

Following cell disruption, the cell powder was resuspended in 400 μL disruption buffer and transferred to a pre-lubricated low bind 1.7 mL tube (BioScience, Inc.). MgCl_2_ (6 mM final conc.) and 12.5 U of Pierce™ Universal Nuclease for Cell Lysis (Thermo Fisher Scientific Inc.) were added, followed by 15 min incubation at 37°C while shaking at 1400 rpm, 5 min incubation in an ultra-sonic bath and subsequent storage at −80°C.

### 2.3 Determination of protein concentration

Protein concentration of whole lysates or purified proteins was determined using the *Micro BCA Protein™ Assay Kit* (Thermo Fisher Scientific Inc.). The bicinchoninic acid (BCA) assay was performed as described by the manufacturer. The buffer used was 20 mM HEPES pH 8 containing 1% (w/v) sodium dodecyl sulfate (SDS). Results were automatically measured using the Synergy H1 Multimode Reader (Agilent Technologies, Inc.) and analyzed using an in-house R script.

### 2.4 Purification of bait and crosslinked PPIs

Purification of SigA-TS and all crosslinked proteins was performed using the C-terminal Twin-Strep-tag of SigA-TS and MagStrep® Strep-Tactin XT beads (IBA Lifesciences GmbH). The purification process followed the manufacturer’s instructions, except the buffers utilized were prepared in-house and differed from the ready-to-use buffers provided by IBA. Our buffer W consisted of 20 mM HEPES with 1% Tween20 (Sigma-Aldrich®) at pH 8. The elution buffer XT additionally contained 100 mM Biotin (IBA Lifesciences GmbH). Purifications were performed using 750 μg of total protein lysate, adjusted to 1% Tween20, while maintaining the smallest possible volume. We used 150 μL of the 5% bead suspension which is equal to 7.5 μL beads. Fusion proteins were bound to the beads by incubating the bead/lysate mixture at room temperature for 1 h on a rotational mixer. After washing three times with buffer W, the bound proteins were eluted with 50 μL of buffer XT for 10 min at 37°C. Immediately afterwards 12.5 μL of 20 mM HEPES pH 8 with 5% SDS (Sigma-Aldrich®) were added for a final SDS concentration of 1% and a final volume of 62.5 μL. Purified proteins were stored at −80°C until further use. Remaining proteins bound by the beads were released by eluting with 20 mM HEPES, pH 8 and 1% SDS at 95°C for 2 min.

### 2.5 SDS-PAGE, Western blot analyses and silver nitrate staining

SDS-PAGEs were performed with NuPAGE™ 4–12% Bis-Tris Midi Protein Gels (Thermo Fisher Scientific Inc.) in a Criterion™ Cell electrophoresis chamber (Bio-Rad Laboratories, Inc.) using NuPAGE™ MES SDS as running buffer, Chameleon® Duo Pre-stained Proteinmaker (LI-COR Environmental) as size marker and 4x Protein Sample Loading Buffer (LI-COR Environmental) with added *β*-mercaptoethanol. Electrophoresis was performed with a PowerPac® 200 (Bio-Rad Laboratories) at 160 V. Silver nitrate staining of gels was executed as described in Shevchenko et al. (1996). For Western blot analyses proteins were blotted onto a Immobilon-FL PVDF (0.45 μm) membrane using the Trans-Blot Turbo Transfer System (Bio-Rad Laboratories, Inc.) as the manufacturer intended (7 min, 2.5 mA, 25 V). Western blots were detected with an in house Strep-Tactin CW800 conjugate (Dr. Alexander Reder, Functional Genomics, University Greifswald) directed against the TS and fluorescence detection was performed with the Odyssey® CLx (LI-COR Environmental) in the 800 nm channel. All results were analyzed with Image Studio™ Lite (LI-COR Environmental).

### 2.6 Crosslink reversal and trypsin/Lys-C digestion

After protein concentration determination samples were incubated at 95°C for 2 h in a ThermoMixer C with ThermoTop (Eppendorf SE) to reverse the cross-linking. An aliquot of 500 ng from each sample was transferred into a 1.7 mL pre-lubricated tube. Sequential Lys-C/trypsin digestion was then performed using hydrophilic Sera-Mag SpeedBeads™ carboxyl magnetic beads (Cytiva, Freiburg, Germany) and hydrophobic Sera-Mag SpeedBeads™ carboxyl magnetic beads (Cytiva). A SP3 beads working solution was then generated as described in Reder et al. (2024). All samples were filled up to the smallest possible volume (20 mM HEPES, 1% (w/v) SDS, pH 8) and combined with 20 μg of beads (1 μL of the working solution). Protein binding, washing and airdrying was carried out as described in Reder et al. (2024), but without automation. Afterwards pellets were resuspended in 8 μL digestion buffer (50 mM HEPES, 1 mM CaCl₂, pH 8), and Lys-C Mass Spec Grade (Promega GmbH) was added at a protease-to-protein ratio of 1:25 (20 ng protease for 500 ng protein). The Lys-C digestion was performed for 3 h at 37°C, with shaking every 1 min and 45 s for 15 s. Following this, 20 ng of Sequencing Grade Trypsin (Promega GmbH) was added, and tryptic digest was conducted overnight at 48°C. The next day, digestion was stopped by adding trifluoroacetic acid to a final concentration of 0.5%. At this stage, 0.3 μL of 10x iRT-Stock (Biognosys AG) along with baker water was added to reach a final volume of 15 μL for the digested samples.

### 2.7 HPLC/MS analysis

Analyses of peptides were performed on a Dionex UltiMate 3,000 RSLC (Thermo Fisher Scientific Inc.) combined with an Orbitrap Exploris™ 480 (Thermo Fisher Scientific Inc.). For this 5 μL of each digested sample were loaded onto an Acclaim™ PepMap™100 C18 pre-column (75 μm ID, 5 μm particle size, 100 Å pore size) (Thermo Fisher Scientific Inc.) with a flowrate of 7 μL/min and 0.1% acetic acid in HPLC-water as loading buffer. Peptides were then separated on an Accucore™ 150-C18 analytical column (25 cm length, 75 μm ID, 2.6 μm particle size, 150 Å pore size) using a binary phase system comprising solvent A (0.1% acetic acid in HPLC-water) and solvent B (100% ACN in 0.1% acetic acid). Separation was achieved using a linear gradient of acetonitrile in 0.1% acetic acid over 30 min at a flow rate of 300 nL/min, with a total run time of 65 min at 40°C.

Peptides were ionized by electrospray ionization (ESI) using a Nanospray Flex™ ion source (Thermo Fisher Scientific Inc.). Data was acquired in data-independent acquisition (DIA) mode. Full MS scans were recorded in the m/z range of 350–1,200 with a resolution of 120,000, and a maximum ion injection time of 60 ms. Higher-energy collisional dissociation (HCD) was employed for peptide fragmentation with a normalized collision energy of 30%. MS/MS spectra were acquired with a resolution of 30,000 across 34 DIA isolation windows of 25 m/z width, with 2 m/z overlap. Fully detailed HPLC and the MS settings can be found in Supplementary material S2 MS settings.

### 2.8 Data analysis

Raw MS files were analyzed sample-wise using the Spectronaut® (version 17) (Biognosys AG) with the directDIA+ (Deep) workflow. Each sample type (4 biological replicates) was searched independently against a custom protein database (Supplementary material S3 MS settings Spectronaut) using trypsin/P as the digestion enzyme with allowance of up to two missed cleavages. This approach was used to prevent the algorithms from attempting to identify peptides in the background simply because they were found in an unrelated sample. Oxidation of methionine was set as variable modification. For peptide identification the precursor Q-value cutoff was set to 0.001 and the protein Q-value cutoff was set to 0.01 for the experiment level and 0.05 for the run level.

All searches were performed in Spectronaut using a two-channel configuration that distinguishes light (^14^N) and heavy (^15^N) precursors within the same DIA run.

#### 2.8.1 Variable modification scheme

Spectronaut’s “Metabolic Labeling > Custom” function was set to treat the heavy channel as a series of variable ^15^N modifications: 15 N(1), 15 N(2), 15 N(3), and 15 N(4). These four options allow the search engine to match any peptide that differs from its light counterpart by 1–4 atomic masses per nitrogen atom, covering the full mass range expected for uniformly labeled proteins. Because ^15^N replaces the backbone and side-chain nitrogens during amino-acid biosynthesis, the modification is not confined to the amide terminus; rather, every nitrogen-containing residue contributes an integer 1 Da shift.

#### 2.8.2 Stable-isotope channel setup

A light-channel spectral library was imported into Spectronaut. Heavy precursors were generated *in silico* by applying the appropriate ^15^N mass shift (15 N(1–4) variable modifications) to every peptide sequence. During DIA extraction both light and heavy precursors were searched against the same 34 × 25 m/z isolation windows that were acquired once per sample; no additional MS2 scans were required. Spectronaut scores every fragment ion without prioritizing any specific ion series. Peptide-centric scoring integrates all matched fragment ions for each channel, providing quantitative Light and Heavy ratios that are exported for downstream ratio analysis. Supplementary Figure 1 shows examples of MS1 XICs for heavy and light isotopoloques of a SigA-TS peptide from one Bait/WT experiment replicate and the same peptide from one Bait/Bait control replicate.

Dynamic mass tolerances were applied for both MS1 and MS2, and maximum intensity-based extraction was used for the precursor signal. Peptides were quantified based on their MS2 area under the curve, with interference correction enabled by excluding multi-channel interferences. Further quantification was not performed with Spectronaut®, but instead with R (R Core Team, 2024) (R version 4.2.1) using various packages tidyverse (Wickham et al., 2019) (v 2.0.0), helfRlein (Gepp et al., 2024) (v 1.5.0), plotly (Sievert, 2020) (v 4.10.4), ggrepel (Slowikowski, 2024) (v 0.9.6), patchwork (Pedersen, 2024) (v 1.3.0), limma (Ritchie et al., 2015) (v 3.60.6), testthat (Wickham, 2011) (v 3.2.1.1). All scripts used can be found in the Supplementary material - Script.

### 2.9 Experimental design and statistical rationale

Sample conditions were categorized into two groups: (i) no stress and (ii) 4% (v/v) EtOH stress, with two sample types (0,2% (v/v) and 0.4% (v/v) FA). A total of 48 samples were measured with the MS. All experiments were performed in biological quadruplicates to be able to assess biological variance, with each biological replicate measured once using LC–MS/MS. Stable isotopic labeling and mass spectrometry were utilized for precise and robust quantification of PPIs. For all MS measurements iRTs (Biognosys AG) were used. Raw MS files were processed with Spectronaut (version 17) and statistical analyses were performed with R. To enhance reliability and minimize experimental variability, the following statistical principles and analyses were applied:

#### 2.9.1 Normalization of isotopic ratios

^14^N and ^15^N isotopic labeling was used to account for variability between biological replicates and ensure accuracy. Mixing differently labeled samples enables normalization by computing light-to-heavy (L/H) ratios. This ratio-based normalization approach improves consistency and reliability, allowing for precise quantification by adjusting each sample’s L/H ratios against calculated normalization factors. Heavy-to-light (H/L) ratios were exported from Spectronaut and inverted (1/H/L) to keep the light (^14^N) channel in the numerator. After ion-level quality filters (Q-value ≤ 0.05, CV ≤ 0.2, ≥ 2 peptides per protein, sequence-coverage ≥ 20%), the median L/H ratio of all remaining peptides in each biological replicate was calculated. Each peptide ratio was divided by its run-specific normalization factor to yield a normalized value, ensuring that the background distribution is centered on 1. Because light and heavy cell pellets were mixed 1:1 before affinity purification, this global-median approach corrects minor mixing errors without requiring a separate “house-keeping” reference. Normalized peptide ratios were then summarized per protein by the median and log2-transformed for differential analysis with limma-eBayes.

#### 2.9.2 Stringent threshold-based filtering

To reduce low-confidence data points, stringent Q-value thresholds and CV limits were applied before normalization. Ion-level filtering included a Q-value cutoff of 0.01 and a CV threshold of the L/H ratio to ensure data robustness. Additionally, proteins with low sequence coverage or proteins identified with less than two unique peptides were filtered out, enhancing the precision of the aggregated protein-level quantification.

#### 2.9.3 Data aggregation and protein level analysis

Aggregation of peptide-level data was performed using the normalized peptide L/H ratios to obtain stable quantification at the protein level. This method mitigates the influence of outliers and improves the reliability of PPI data across replicates.

#### 2.9.4 Statistical analysis using the Limma package

The limma package was applied to log_2_-transformed ratio data to assess differential protein abundance across conditions. The empirical Bayes test method was utilized on log_2_ ratios. Multiple testing correction was performed using the Benjamini-Hochberg (BH) (Benjamini and Hochberg, 1995) adjustment within the *topTable* function and BH adjusted *p*-values (adj. P. Val) were computed for all proteins, and significance was defined as adj. P. Val ≤ 0.001 (FDR ≤ 0.1%) controlling the false discovery rate (FDR) and ensuring high-confidence identification of significant interactions.

This statistical framework, incorporating limma’s moderated statistical testing alongside rigorous filtering and normalization, ensures reliable identification and quantification of PPIs by minimizing experimental variability and excluding low-confidence data points. The approach enhances data robustness and supports the high confidence necessary for accurate biological insights.

## 3 Results

**Figure 2:**
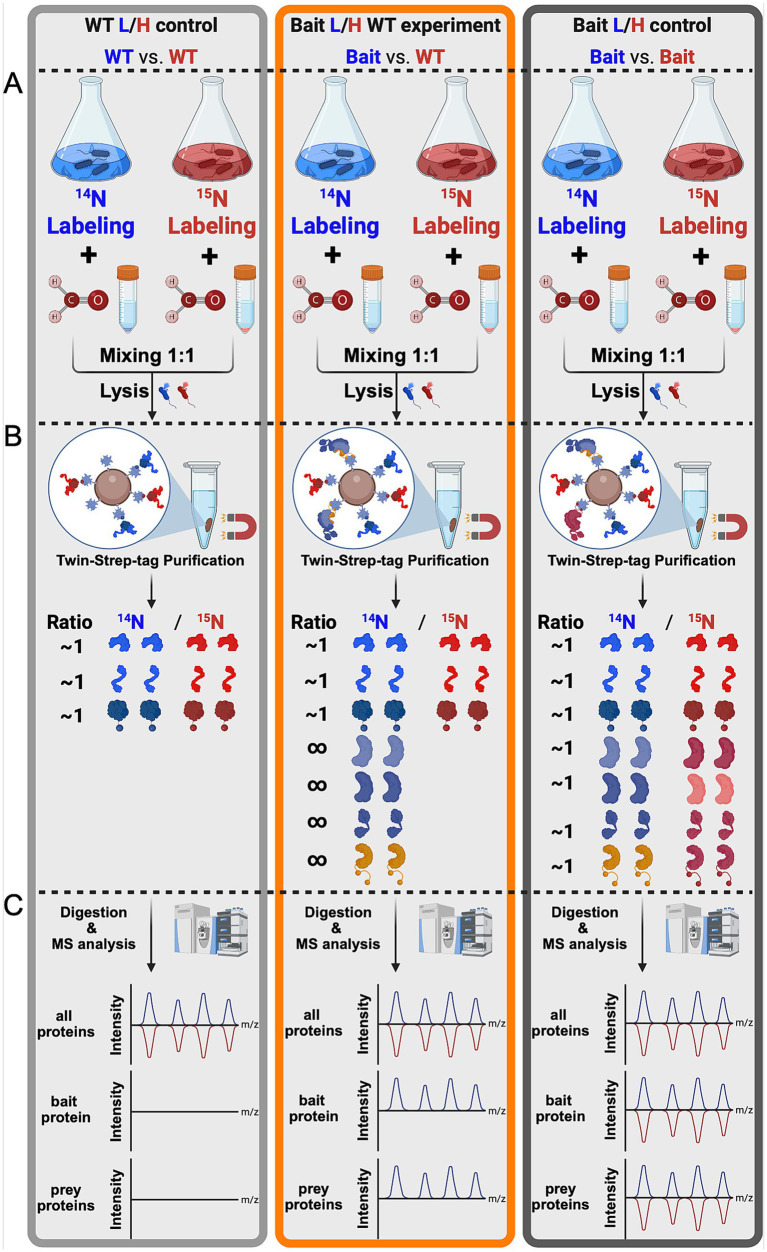
TIE-UP-SIN general workflow. Schematic presentation of the TIE-UP-SIN workflow split into three sections **(A–C)**. Differential isotope labeling (^14^N/^15^N), cross linking, mixing of equal cell numbers of the control and bait strain according to the scheme WT/WT (light gray), Bait/WT (orange) and Bait/Bait (dark gray) and cell lysis in Section **A**. Followed by affinity purification of the Bait protein via the Twin-Strep-tag and reversal of formaldehyde crosslinks in Section **B**. As well as sample digestion, MS analysis and theoretical results of the mass spectrometric analysis in Section **C**.

Here, we developed the TIE-UP-SIN (Targeted Interactome Experiment for Unknown Proteins by Stable Isotope Normalization) approach to improve the detection and quantification of protein–protein interactions (PPIs) in an *in vivo* setting. The general workflow, as shown in the Figure 2, integrates stable metabolic isotope labeling, rigorous controls, reversible cross-linking, and affinity purification coupled with highly sensitive MS to improve the robustness and accuracy of PPI identification without *a priori* knowledge of potential PPIs. We applied TIE-UP-SIN to study the known PPIs of SigA, the essential housekeeping sigma factor from *B. subtilis* and assessed the effectiveness and robustness of the newly developed TIE-UP-SIN method. The workflow (Figure 2) includes three different sample setups, WT L/H control, Bait L/H WT experiment and Bait L/H control, which were handled identically from cell lysis through purification and MS analysis.

### 3.1 General workflow

To establish TIE-UP-SIN, we first generated the bait strain from the wild-type (WT) strain. The coding sequence for a Twin-Strep-tag was chromosomally fused to the 3’end of the *sigA* gene of the WT strain (BSB1) at the native chromosomal location, as illustrated in Figure 1. Following this modification, the WT and bait strain were cultivated in both, ^14^N and ^15^N BioExpress media, as described in the *Experimental Procedures* section. Samples for TIE-UP-SIN were collected under standard exponential growth conditions and 10 min after the exposure EtOH stress (4% (v/v) final concentration) to evaluate interaction profiles under both conditions.

The prolonged exposure to labeled medium allowed for quantitative incorporation of nitrogen isotopes into cellular proteins, ensuring high labeling efficiency (>98%). The reliable isotopic labeling across all experimental conditions supports (1) robust and quantitative PPI analysis, (2) enables precise and quantitative comparison between different biological conditions, (3) reflects *in vivo* physiological protein levels and modifications, (4) minimizes sample variability due to sample preparation as well as instrument conditions and most importantly (5) allows for quantification through relative intensity ratios of light and heavy peptides in MS.

Formaldehyde was used to crosslink the cultures during harvesting. Due to its small molecular size, formaldehyde efficiently penetrates cell walls and membranes and enables rapid cross-linking of primary amines in neighboring proteins (2–3 Å) (Sutherland et al., 2008), facilitating the maintenance of in *vivo* interactions. To ensure optimal cross-linking without excessive modification, we tested two different concentrations of formaldehyde, 0.2% as well as 0.4% (w/v). This approach allowed us to capture relevant protein interactions efficiently while minimizing the risk of over-crosslinking, which can complicate the downstream purification process by inactivation of the affinity purification tag.

#### 3.1.1 Cell mixing experiment

Prior to cell lysis, light-labeled and heavy-labeled cell pellets were combined to prepare the samples for subsequent analysis. Table 1 presents the combinations of pellets, and the resulting samples generated from these combinations.

Following the purification of all samples using Twin-Strep-tag magnetic beads, distinct outcomes were anticipated for each sample:

##### 3.1.1.1 WT/WT control sample

In this control sample, equal amounts (OD units) of light-labeled and heavy-labeled WT pellets were mixed. Since the WT strain lacks a Twin-Strep-tag on *sigA*, only proteins that non-specifically bind to the TactinXT on the magnetic beads (used for purifying TS-tagged proteins) should be present in the eluate in a L/H ratio around 1. The purpose of the WT control sample is to identify these non-specifically purified proteins, establishing a baseline for non-specific binding in the experiment. From here on this sample will be called WT/WT or WT/WT control.

##### 3.1.1.2 Bait/WT experiment

In this experimental sample, light-labeled SigA-tagged cells were combined with heavy-labeled WT cells. Since in the Bait strain *sigA* is fused to a Twin-Strep-tag, SigA-TS itself and all proteins cross-linked to it are expected to be purified and thus strongly enriched, resulting in a L/H ratio greater than 1. This is illustrated by the infinity symbol in Figure 1, Section B. All non-specific proteins identified in the WT control sample should also be present in this sample, with a L/H ratio around 1. The comparison with the WT control sample enables us to differentiate specific SigA interactors, co-eluted during purification, from non-specific purified non-interactor proteins. From here on this sample will be referred as Bait/WT or Bait/WT experiment.

##### 3.1.1.3 Bait/Bait control sample

For this control, light-labeled and heavy-labeled SigA-TS-tagged cells were mixed. Since both strains express TS-tagged SigA, the expected outcome after purification is an even distribution of proteins across the light and heavy channels. The ^14^N/^15^N ratio should be around 1 for all proteins, including SigA and any associated interactors. This control serves to validate that the observed enrichments in the Bait/WT experiment sample are due to specific interactions with SigA, rather than artifacts or background binding caused by the purification tag. Additionally, it also serves as comparison of growth in the two differently labeled media. From here on this sample will be called Bait/Bait or Bait/Bait control.

### 3.2 Crosslinking validation

**Figure 3:**
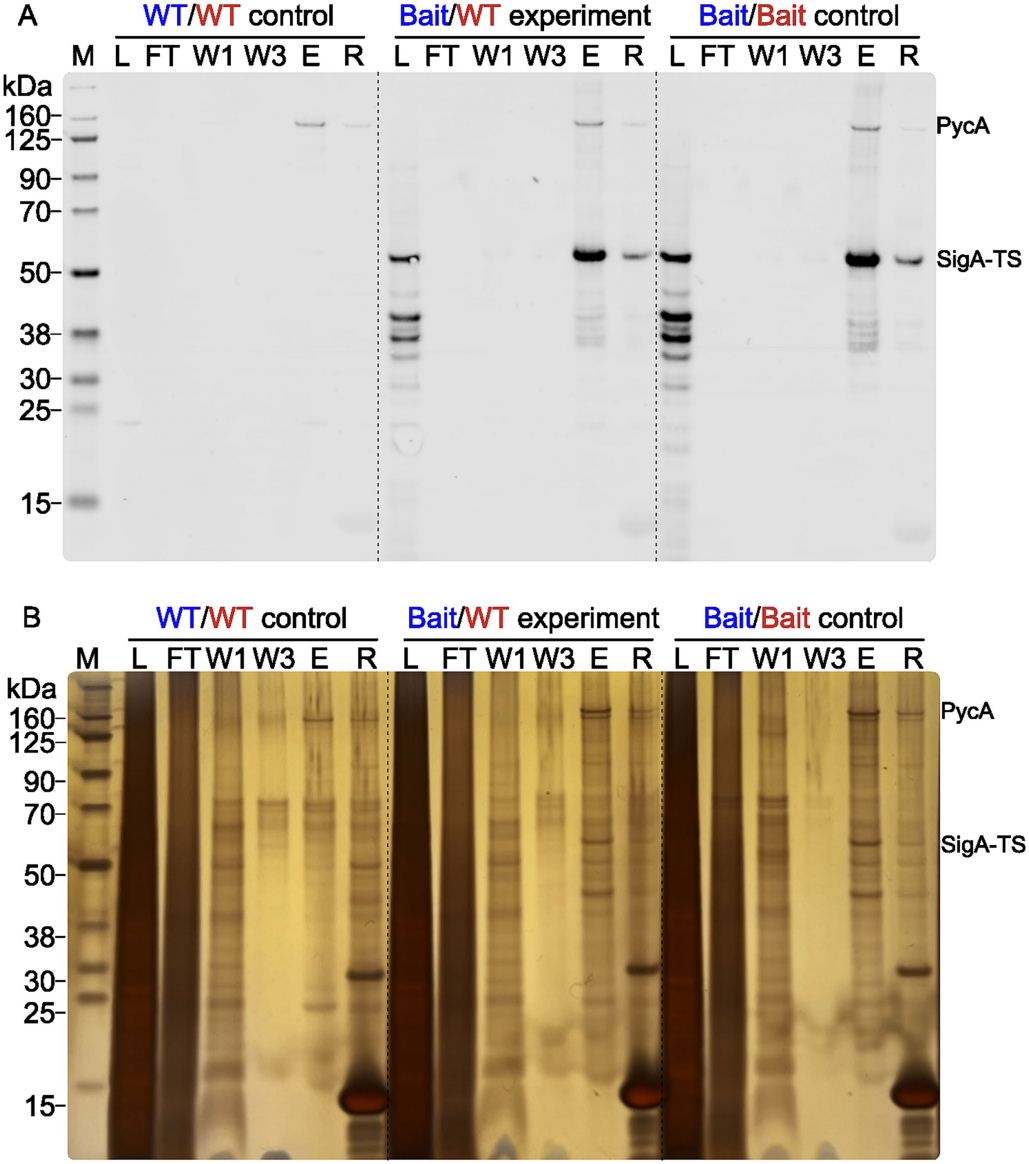
Purification validation by Western blot and silver gel analysis. Western blot **(A)** and silver-stained gel **(B)** show the purification fractions of the WT control (WT/WT), Bait/WT experiment (Bait/WT), and Bait control (Bait/Bait), all crosslinked with 0.2% (w/v) FA. The fractions loaded are lysate (L) (1 μL), flowthrough (FT) (1 μL), wash steps 1 (W1) and 3 (W3) (8 μL), eluate (E) (4 μL), and residue (R) (4 μL). The Chameleon® Due Pre-stained Protein Marker was used for molecular weight reference. The position of the signals for PycA (127.72 kDa) and SigA-TS (45.9 kDa) are marked on the right side.

The purification was validated with a Western blot analysis and an analysis of silver nitrate stained gels. In Figure 3 a Western blot (3A) and a silver nitrate stained gel (3B) of one replicate from the 0.2% (w/v) formaldehyde samples are shown as an example of purification validation. All samples were heated at 95°C for 2 h before loading onto an SDS-PAGE to resolve cross-links. The Western blot shows the expected results with only one apparent signal in the eluate (E) lane of the WT/WT control at around 130 kDa. This later turned out to be one of the expected unspecifically purified proteins, namely the pyruvate carboxylase (PycA), which was present in all samples in similar abundance. PycA was unspecifically purified to that extent because it is biotinylated and the Tactin-XT capture protein on the magnetic purification beads also binds biotin (Henke and Cronan, 2014). The Bait/Bait control and the Bait/WT experiment both show substantial amounts of purified SigA-TS (45.9 kDa), as expected. The silver nitrate stained gel presents a first opportunity to screen for putative crosslinked and copurified proteins, particularly if additional signals were present in the eluates of the Bait/WT experiment and Bait/Bait control compared to the WT/WT control. This visual inspection was the first of two to identify usable samples for the digest and MS measurement. This is recommended to prevent further working with unusable samples. For example, if the reversal of the FT crosslinks was not complete. Before sample digestion and MS analysis, the formaldehyde crosslinks needed to be reversed. The success of this was again validated via Western blot analysis to avoid unnecessary MS measurements of flawed samples. Exemplary Western blots for all unstressed samples are shown in the Supplementary Figure 2. Elution fractions are presented both prior to and following the reversal of crosslinking. The room temperature lanes (RT) all show the purified crosslinked proteins at the top of the blot. Due to the crosslinked high molecular weight complexes their size is too big as they could not easily enter and move through the gel during electrophoresis.

### 3.3 Enrichment analysis

For the data analysis, we established specific filtering parameters, which are detailed in Table 2 alongside brief descriptions and the initial standard values for a stringent filtering. During the analysis, all protein data were normalized at the peptide level using computed normalization factors for each peptide in each sample. The peptide-level data was then aggregated to the protein level as described in methods. The initial filter settings serve as the starting point, from which ion_CV and seqcov were adjusted and iterated until *sigA-TS* was included. The ion_CV parameter is a threshold for the light/heavy ion ratio CV and the seqcov parameter is a threshold for protein sequence coverage. The specific adjustments may vary slightly for each experiment. In Supplementary Figures 3, 4 we performed the enrichment analysis with the same filter parameters for all experiments. The resulting volcano plots show that for every experiment the best fitting set of filter parameters has to be determined. For independent exploration and modification of filtering parameters, the data sets and analysis pipeline are available in an R Shiny app under the URL: https://shiny-fungene.biologie.uni-greifswald.de/TIE_UP_SIN_app (login: reviewer_login; password: Kr3Tjuji? Hfsilh). Readers are encouraged to try changing the filter parameters on their own and explore the results.

**Table 2:** Filter parameters, a short description and their initial values for the TIE-UP-SIN data analysis.

TIE-UP-SIN filter parameters		
Parameter name	Description	Initial
Control_FC	Peptides from controls with dissimilar L/H intensities are excluded	2
Q. Value	Q.-Value must be greater	0.01
ion_CV	Ion CVs for each condition must be smaller	0.2
DataCompletness	Proteins must be detected in all replicates	1
Seq-Coverage	Sequence coverage (%) per protein must be greater	40
NumPeptides	At least 2 peptides are required per protein	2

If parameters are too strict, increasing the ion_CV cut off is the first step to less stringent filtering.

#### 3.3.1 Enrichment data for 0.2% FA under control conditions

**Figure 4:**
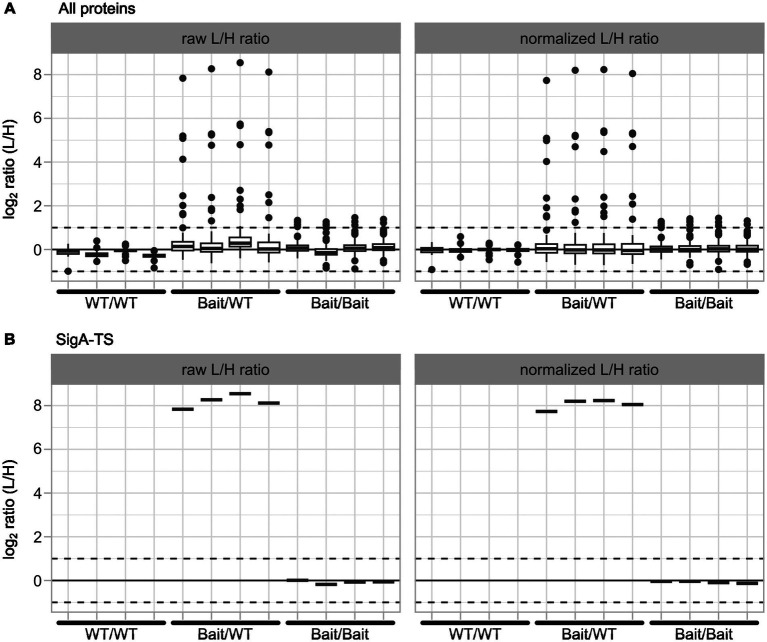
Normalization results for all proteins and SigA-TS. The plots present log_2_ values of raw L/H ratios (left panels) and normalized L/H ratios (right panels). Data are shown for all proteins **(A)** and for SigA-TS **(B)**. Each panel displays the four biological replicates for the three samples: WT/WT, Bait/WT, and Bait/Bait, arranged sequentially from left to right along the x-axis. All L/H ratio values are transformed to log_2_ scale for consistent representation.

The normalization results for all proteins and SigA-TS for one of the data sets are shown in Figure 4. Overall, the normalization effect on all proteins (Figure 4A) was minimal, as most proteins initially displayed an L/H ratio close to 1. As expected, the Bait/WT sample, some proteins demonstrated strong enrichment in the light channel, suggesting they are potential interaction partners of SigA-TS. Figure 4B illustrates the normalization effect on SigA-TS. As anticipated, SigA-TS exhibited distinct L/H ratios across the three sample types: no presence in the WT/WT control replicates, high L/H ratios in the Bait/WT experiment replicates, and L/H ratios around 1 in the Bait/Bait control replicates.

**Figure 5:**
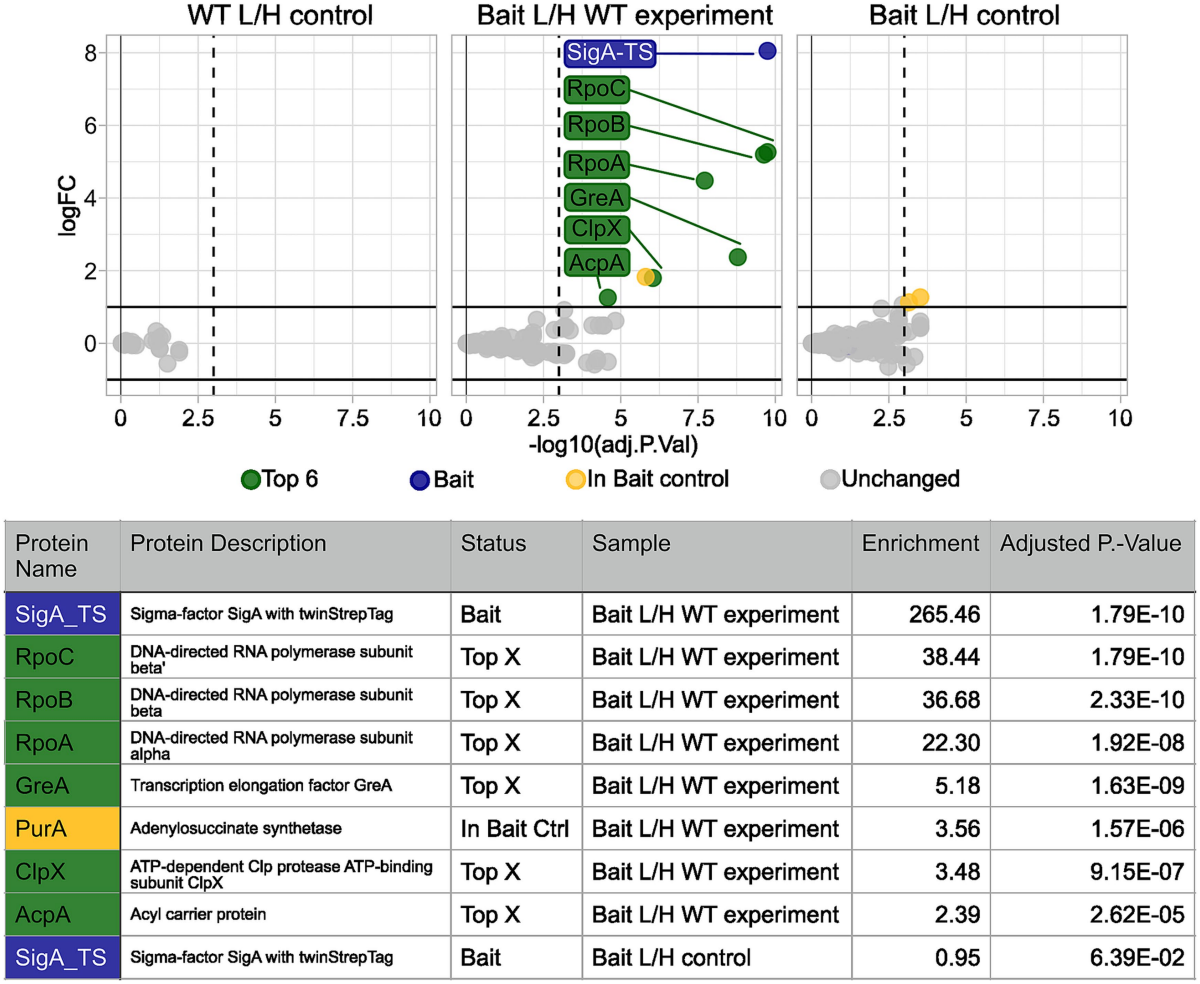
Calculated protein enrichment from MS data – control condition experiment. Results for the SigA-TS crosslinked with 0.2% (w/v) formaldehyde. Initial filter parameters were used. Volcano plots depict the log_2_ enrichment of proteins in the WT L/H control (left), the Bait/WT experiment (middle), and the Bait L/H control (right). The x-axis shows the log_2_ fold enrichment, and the y-axis indicates statistical significance (−log_10_ adjusted *P*-Value). Gray dots represent detected but L/H ratio wise unchanged proteins, while colored markers highlight significantly enriched proteins based on the adjusted *p*-value threshold (vertical dashed line) and the enrichment threshold (horizontal solid line). Highlighted proteins, such as SigA-TS (blue), RpoC, RpoB, RpoA, and GreA (green) met these thresholds and are labeled. Below the plots, a summary table details the names, descriptions, status, sample, and adjusted *p*-values of the highlighted proteins.

Figure 5 illustrates an example of the data analysis results generated using the TIE-UP-SIN analysis, presented as a volcano plot together with the corresponding table detailing the identified interactors and their respective enrichment values (fold changes). This example uses a sample set treated with 0.2% (w/v) formaldehyde under control conditions with the filtering parameters ion_CV and seqcov adjusted to 0.3 and 30, respectively. The resulting volcano plot reveals no significantly enriched proteins in both the WT/WT as well as the Bait/Bait control samples. As expected, SigA-TS is absent in the WT/WT control and exhibits an L/H ratio close to 1 in the Bait/Bait control. In contrast, the Bait/WT experiment sample demonstrated the highest enrichment factor of the SigA-TS bait protein with a 265-fold increase compared to the wild-type control. This exceptional enrichment was statistically significant, with an adjusted *p*-value of 1.79 × 10^−10^, underscoring the high specificity and efficacy of the TIE-UP-SIN approach in detecting and quantifying protein interactions.

The primary interaction partners of SigA, RpoB, RpoC, and RpoA which are part of the core RNA polymerase complex, exhibited high enrichment factors of 38, 37, and 22, respectively, with significant adjusted *p*-values. Notably, the closely matched enrichment factors of RpoB and RpoC align with their similar binding affinities to SigA and their spatial proximity in the RNA polymerase (RNAP) complex (Bae et al., 2015). These findings validate the known role of SigA in the context of RNAP and transcription initiation. Another well-known RNAP interaction partner and secondary SigA interactor, GreA (Laptenko et al., 2003), was detected with a lower enrichment factor of 5.2 and an adjusted p-value of 1.63 × 10^−9^.

Beyond these well-characterized interactions, the TIE-UP-SIN method also uncovered potential non-canonical interactors, including ClpX and AcpA, broadening the understanding of SigA’s functional network.

PurA was identified in the experiment with an enrichment factor of 3.6. However, since it was also detected in the bait control sample, it is highlighted in yellow and excluded from the list of potential positive interactors. Interestingly, ClpX, the ATPase subunit of the ATP-dependent ClpXP protease involved in protein quality control and regulatory processes (Krüger et al., 2000), was also identified with an enrichment factor of 3.5 and an adjusted p-value of 9.15 × 10^−7^. AcpA, the acyl carrier protein central to fatty acid biosynthesis, was detected with an enrichment factor of 2.4 and an adjusted p-value of 2.62 × 10^−5^. While its interaction with SigA is unexpected, this finding might reflect a functional link between lipid metabolism and transcriptional regulation. Compared to the RNAP subunits, which showed enrichment factors exceeding 20-fold, the more modest enrichments observed for ClpX and AcpA underscore the remarkable sensitivity of TIE-UP-SIN. This sensitivity enables the detection of lower abundance or transient interactions.

When we applied less stringent parameters, specifically by decreasing the sequence coverage threshold to 20%, we identified eight additional proteins (Supplementary Figure 5). Notably, this included the remaining RNAP components RpoE and RpoY, which were not detected under more stringent conditions. While proteins with low enrichment and low adjusted *p*-values may not represent direct or indirect interaction partners of SigA, most of the newly identified proteins are known to be in close spatial proximity to the RNAP complex or involved in transcription or translation processes.

#### 3.3.2 Comparison of 0.2 and 0.4% FA

To optimize cross-linking conditions for the TIE-UP-SIN method, we compared the performance of 0.2% (w/v) and 0.4% (w/v) formaldehyde (FA) concentrations. Overall, the results were consistent between the two FA concentrations, with similar enrichment patterns observed. However, the higher FA concentration had a notable drawback: reduced purification yield. This could be attributed to a higher extent of cross-linking at 0.4% (w/v) FA, potentially leading to a cross-linked tag unusable for purification. Additionally, higher FA concentrations could increase non-specific cross-linking, potentially introducing additional background and false positives. Conversely, the higher FA concentration likely improved cross-linking of the bait protein and its interactors, leading to more robust enrichment.

In summary, while both FA concentrations yielded comparable results under optimized filtering conditions, 0.4% (w/v) FA provided a greater confidence in statistical results. In contrast, 0.2% (w/v) FA resulted in less non-specific cross-linking and lower background. The choice of FA concentration should therefore balance recovery efficiency, statistical robustness, and the need to minimize potential cross-linking artifacts, depending on the experimental goals. We recommend testing both concentrations when investigating a protein for the first time, particularly if the protein has only a few potential interaction partners.

#### 3.3.3 Comparison of control condition and 4% EtOH stress

**Figure 6:**
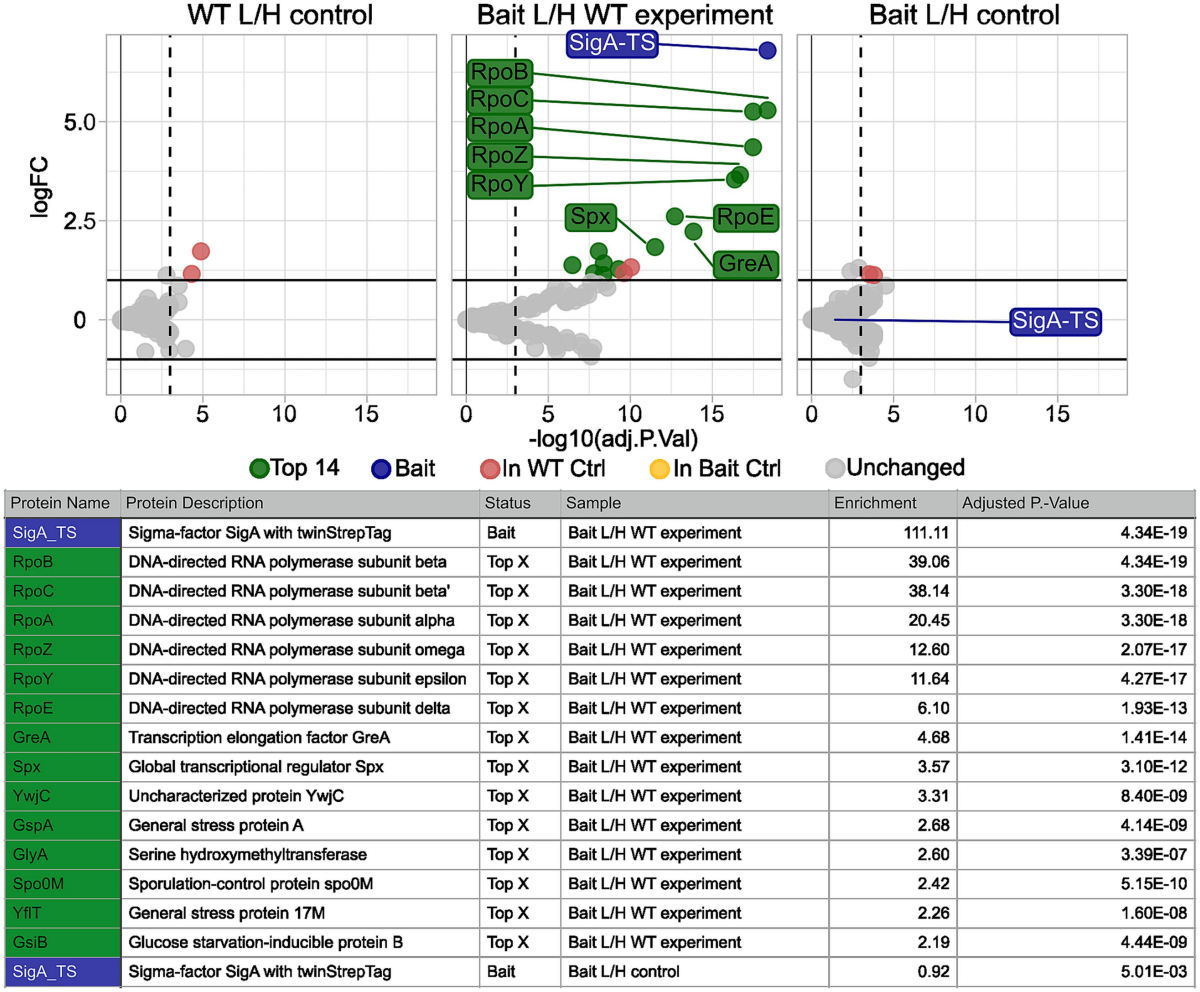
Calculated protein enrichment from MS data – 4% EtOH stress experiment. Results for the SigA-TS under 4%(v/v) EtOH stress and crosslinked with 0.4% (w/v) formaldehyde. Initial filter parameters were used except ion_CV which was set to 0.3. Volcano plots depict the log_2_ enrichment of proteins in the WT L/H control (left), the Bait/WT experiment (middle), and the Bait L/H control (right). The x-axis shows the log_2_ fold enrichment, and the y-axis indicates statistical significance (−log_10_ adjusted P-Value). Gray dots represent detected but L/H ratio wise unchanged proteins, while colored markers highlight significantly enriched proteins based on the adjusted p-value threshold (vertical dashed line) and the enrichment threshold (horizontal solid line). Highlighted proteins, such as SigA-TS (blue), RpoC, RpoB, RpoA, and GreA (green) met these thresholds and are labeled. Yellow and red dots indicate proteins that exceeded the thresholds but were enriched in one of the two control samples and thus were excluded from the list of potential interaction partners. Below the plots, a summary table details the names, descriptions, status, sample, and adjusted p-values of the highlighted proteins.

We conducted the TIE-UP-SIN experiment with SigA-TS as bait under 4% (v/v) ethanol stress, using 0.2 and 0.4% (w/v) formaldehyde for cross-linking. The results for 0.2% (w/v) FA are presented in Figure 6. Under these stress conditions, we identified the same interaction partners of SigA as in the control condition and additional interactors unique to the stressed condition. For protein identification, the ion coefficient of variation (ion_CV) was set to 0.3 to ensure robust detection of SigA-TS in the samples. The results are shown in Figure 6. As anticipated, SigA-TS showed strong enrichment in the Bait/WT experiment with a log_2_ enrichment value of 195, a near 1 L/H ratio in the Bait/Bait control (0.93) and was absent in the WT/WT control. Among the top enriched proteins in the Bait/WT experiment were RpoB, RpoC, and RpoA, with enrichments of 40, 39.5 and 18.6, respectively. These proteins are well-established direct interaction partners of SigA. Secondary interaction partners of SigA, including RpoZ, RpoY, RpoE and GreA, also showed significant enrichment, with values of 12.60, 11.6, 6.1 and 4.7, respectively.

Additionally, Spx displayed a moderate enrichment of 3.6. Spx has been recognized as a direct SigA and RpoA interactor before (Zuber, 2004; Nakano et al., 2010). Spo0M (Vega-Cabrera et al., 2017), has also been enriched, but is not known to interact directly with SigA so far.

Several members of the SigB regulon,including YwjC, GspA, G17M (YflT) and GsiB showed a slight enrichment (above 2-fold) in the ethanol stressed sample. While this enrichment suggests that these proteins were captured during the crosslinking procedure, it remains unclear whether this reflects a specific or functional association with SigA. Additional proteins that were also enriched in either the WT/WT control or the Bait/Bait control were excluded from the list of potential direct SigA interaction partners, which highlights the value of the inclusion of the additional controls. These proteins are highlighted in yellow and red in the volcano plots in Figure 6.

## 4 Discussion

The TIE-UP-SIN method demonstrated high sensitivity and specificity in capturing both known and novel interaction partners of SigA. The significant enrichment of RNAP core subunits validates the reliability of the approach. Isotopic labeling allowed precise quantification under native conditions, and formaldehyde cross-linking preserved transient interactions.

The enrichment of ClpX suggests a possible regulatory link between SigA and proteolytic systems. ClpX may influence SigA stability indirectly by targeting cofactors or repressors. Similarly, the detection of AcpA, a central protein in fatty acid biosynthesis, may indicate coordinated regulation between membrane synthesis and transcription. These non-canonical interactors highlight the potential of TIE-UP-SIN to uncover previously unknown associations, whether direct or proximity-based.

The flexibility of the filtering strategy proved valuable: while stringent filters yielded high-confidence hits, relaxed settings uncovered spatial neighbors or low-affinity partners (e.g., RpoY, RpoE). Nonetheless, lowering thresholds increases the risk of non-specific identifications; hence, cautious interpretation is advised.

The comparison of FA concentrations showed that 0.2% FA yields cleaner eluates with fewer background binders, whereas 0.4% FA may increase cross-linking efficiency but at the cost of recovery. We recommend testing both conditions when establishing a new target, particularly for low-abundance proteins.

The application of TIE-UP-SIN under ethanol stress revealed stress-induced changes in the SigA interactome. While core RNAP subunits remained enriched, additional proteins such as Spx and Spo0M were detected. For Spx, it is well established that, upon induction by ethanol and activation by oxidative stress, it interacts with the C-terminal domains of the RNA polymerase *α*-subunits (Newberry et al., 2005; Petersohn et al., 1999; Nakano et al., 2003). In contrast, Spo0M has not been previously associated with RNAP interaction; its detection may reflect a sporulation-priming response or a stress-related proximity effect, warranting further investigation. Moderate enrichment of general stress proteins from the SigB regulon is likely due to transient proximity rather than direct binding, highlighting the importance of proper controls.

We adopted uniform heavy-nitrogen (^15^N) metabolic labeling because it lets the light (^14^N) and heavy (^15^N) cultures be mixed even before cell disruption, embedding a 1:  1 internal reference that accompanies every wash, cross-link-reversal and digestion step. Expressing the data as heavy-to-light ratios within the *same* chromatogram removes run-level artifacts - column loading, ionization, detector drift - that intensity-based methods must correct post-hoc. Because *B. subtilis* grows prototrophically in minimal medium, replacing NH₄Cl with ^15^NH₄Cl achieves ≥ 98% incorporation after one passage; the medium surcharge adds to the LC–MS budget, however is offset by halving instrument time relative to peptide-level tags such as TMT or dimethyl, which still require separate pull-downs for every channel.

A theoretical concern with metabolic labeling is mass-dependent ionization bias, which is often mitigated by running label-swapped (reciprocal) replicates. In our hands the baseline light/heavy distribution of non-enriched proteins was centered on 1. Because heavy and light lysates are mixed prior to affinity capture, any purification artifact applies equally to both channels; a reciprocal design would therefore double instrument time without measurably improving quantitative accuracy (Oda et al., 1999).

TIE-UP-SIN is compatible with antibody capture where genetic tagging is not feasible. The essential safeguards are unchanged: isotype and bead-only controls, and - where possible - knockout/epitope-deleted controls to verify specificity. Because formaldehyde can mask epitopes, FA should be titrated (we used 0.2–0.4%) and target recovery confirmed after cross-link reversal. Isotopic labeling is not strictly required (label-free or peptide-tagged variants are possible), but we recommend uniform ^15^N labeling wherever feasible: pre-capture mixing equalizes losses between channels, ratio-metric quantification in a single chromatogram minimizes batch effects, and MS time is reduced compared with peptide-level tags. In *Bacillus subtilis*, ≥ 98% incorporation is achieved after one passage in minimal medium. If labeling is impractical, robust controls, replicate randomization, appropriate normalization and conservative thresholds are essential to avoid over-calling low-stoichiometry or proximity contaminants.

Taken together, the TIE-UP-SIN approach enables the robust identification of primary interaction partners with high confidence. Additional proteins detected under relaxed filtering conditions or stress-induced states likely represent secondary or tertiary interactors, reflecting indirect associations or spatial proximity. Their biological relevance should therefore be interpreted with caution and validated through complementary methods.

## 5 Conclusion

The novel TIE-UP-SIN methodology successfully identifies known, specific PPIs of SigA and provides high-confidence data for the analysis of protein interaction networks in *Bacillus subtilis*. By combining *in vivo* formaldehyde crosslinking, stable isotope labeling with ^15^N, and quantitative mass spectrometry, TIE-UP-SIN enables the detection of both stable and transient interactions - including those that are often missed by traditional affinity purification or proximity-labeling approaches. The internal L/H ratio-based quantification ensures precise measurement of interaction partners by reduction of experimental variability and improved reproducibility across biological replicates.

Unlike classical SILAC approaches, which are challenging to implement in *B. subtilis* due to its prototrophic nature and complex nitrogen metabolism, TIE-UP-SIN employs global ^15^N metabolic labeling. This circumvents the need for engineered auxotrophic strains and allows for efficient and reproducible isotopic incorporation using commercially available media. As a result, TIE-UP-SIN establishes a practical and robust route to quantitative interactomics in *B. subtilis*, and is well-suited for broader application in non-model organisms or environmental isolates with limited genetic tractability.

The use of customizable filtering parameters enhances the statistical robustness of interaction data analysis, enabling researchers to fine-tune the balance between sensitivity and stringency based on specific experimental objectives. Although the generation of a Twin-Strep-tagged bait protein is a prerequisite, this requirement is outweighed by the method’s overall simplicity, scalability, and compatibility with physiological *in vivo* conditions. Importantly, the detection of both well-established SigA interactors and previously uncharacterized candidates highlights the sensitivity and discovery potential of TIE-UP-SIN. Although functional validation of these novel interactors lies beyond the scope of this methodological study, their identification underscores the workflow’s capacity to generate biologically meaningful hypotheses. Notably, TIE-UP-SIN is currently being applied successfully to investigate the interactomes of proteins with entirely unknown functions, further demonstrating its utility in exploratory proteomics.

In summary, TIE-UP-SIN represents a reliable, adaptable, and cost-effective platform for studying protein–protein interactions in bacteria. Its methodological flexibility, combined with robust quantification and physiological relevance, makes it a valuable addition to the proteomic toolbox for investigating dynamic protein networks across diverse biological contexts.

## Data Availability

The original contributions presented in the study are publicly available. This data can be found at: https://massive.ucsd.edu/, MSV000097686.

## References

[ref1] Abdiche Y. N. Malashock D. S. Pinkerton A. Pons J. (2009). Exploring blocking assays using octet, ProteOn, and Biacore biosensors. Anal. Biochem. 386, 172–180. doi: 10.1016/j.ab.2008.11.038, PMID: 19111520

[ref2] Abramson J. Adler J. Dunger J. Evans R. Green T. Pritzel A.. (2024). Accurate structure prediction of biomolecular interactions with AlphaFold 3. Nature 630, 493–500. doi: 10.1038/s41586-024-07487-w, PMID: 38718835 PMC11168924

[ref3] Bae B. Feklistov A. Lass-Napiorkowska A. Landick R. Darst S. A. (2015). Structure of a bacterial RNA polymerase holoenzyme open promoter complex. eLife 4:e08504. doi: 10.7554/eLife.08504, PMID: 26349032 PMC4593229

[ref4] Benjamini Y. Hochberg Y. (1995). Controlling the false discovery rate: a practical and powerful approach to multiple testing. J. R. Stat. Soc. Ser. B Methodol. 57, 289–300. doi: 10.1111/j.2517-6161.1995.tb02031.x

[ref5] Collins K. M. Evans N. J. Torpey J. H. Harris J. M. Haynes B. A. Camp A. H.. (2023). Structural analysis of *Bacillus subtilis* sigma factors. Microorganisms 11:1077. doi: 10.3390/microorganisms11041077, PMID: 37110501 PMC10141391

[ref6] Fields S. Song O. (1989). A novel genetic system to detect protein–protein interactions. Nature 340, 245–246. doi: 10.1038/340245a0, PMID: 2547163

[ref7] Gavin A. C. Bösche M. Krause R. Grandi P. Marzioch M. Bauer A.. (2002). Functional organization of the yeast proteome by systematic analysis of protein complexes. Nature 415, 141–147. doi: 10.1038/415141a, PMID: 11805826

[ref8] Gepp J. Luettgau D. Bleier A. Krabel T. Albers M. Feick L. (2024). *helfRlein: R Helper Functions*. https://github.com/STATWORX/helfRlein

[ref9] Gingras A. C. Gstaiger M. Raught B. Aebersold R. (2007). Analysis of protein complexes using mass spectrometry. Nat. Rev. Mol. Cell Biol. 8, 645–654. doi: 10.1038/nrm2208, PMID: 17593931

[ref10] Grajales E. J. Alarcón E. A. Villa A. L. (2015). Kinetics of depolymerization of paraformaldehyde obtained by thermogravimetric analysis. Thermochim. Acta 609, 49–60. doi: 10.1016/j.tca.2015.04.016

[ref11] Haldenwang W. G. (1995). The sigma factors of *Bacillus subtilis*. Microbiol. Rev. 59, 1–30. doi: 10.1128/mr.59.1.1-30.1995, PMID: 7708009 PMC239352

[ref12] Henke S. K. Cronan J. E. (2014). Successful conversion of the *Bacillus subtilis* BirA group II biotin protein ligase into a group I ligase. PLoS One 9:e96757. doi: 10.1371/journal.pone.0096757, PMID: 24816803 PMC4016012

[ref13] Herzberg C. Weidinger L. A. F. Dörrbecker B. Hübner S. Stülke J. Commichau F. M. (2007). SPINE: a method for the rapid detection and analysis of protein-protein interactions in vivo. Proteomics 7, 4032–4035. doi: 10.1002/pmic.200700491, PMID: 17994626

[ref14] Johnston E. B. Lewis P. J. Griffith R. (2009). The interaction of *Bacillus subtilis* σA with RNA polymerase. Protein Sci. 18, 2287–2297. doi: 10.1002/pro.239, PMID: 19735077 PMC2788283

[ref15] Jumper J. Evans R. Pritzel A. Green T. Figurnov M. Ronneberger O.. (2021). Highly accurate protein structure prediction with AlphaFold. Nature 596, 583–589. doi: 10.1038/s41586-021-03819-2, PMID: 34265844 PMC8371605

[ref16] Krüger E. Witt E. Ohlmeier S. Hanschke R. Hecker M. (2000). The Clp proteases of *Bacillus subtilis* are directly involved in degradation of misfolded proteins. J. Bacteriol. 182, 3259–3265. doi: 10.1128/JB.182.11.3259-3265.2000, PMID: 10809708 PMC94515

[ref17] Laptenko O. Lee J. Lomakin I. Borukhov S. (2003). Transcript cleavage factors GreA and GreB act as transient catalytic components of RNA polymerase. EMBO J. 22, 6322–6334. doi: 10.1093/emboj/cdg610, PMID: 14633991 PMC291851

[ref18] Liedberg B. Nylander C. Lunström I. (1983). Surface plasmon resonance for gas detection and biosensing. Sensors Actuators 4, 299–304. doi: 10.1016/0250-6874(83)85036-7

[ref19] MacBeath G. Schreiber S. L. (2000). Printing proteins as microarrays for high-throughput function determination. Science 289, 1760–1763. doi: 10.1126/science.289.5485.1760, PMID: 10976071

[ref20] Nakano M. M. Lin A. Zuber C. S. Newberry K. J. Brennan R. G. Zuber P. (2010). Promoter recognition by a complex of Spx and the C-terminal domain of the RNA polymerase α subunit. PLoS One 5:e8664. doi: 10.1371/journal.pone.0008664, PMID: 20084284 PMC2801614

[ref21] Nakano S. Nakano M. M. Zhang Y. Leelakriangsak M. Zuber P. (2003). A regulatory protein that interferes with activator-stimulated transcription in bacteria. Proc. Natl. Acad. Sci. USA 100, 4233–4238. doi: 10.1073/pnas.0637648100, PMID: 12642660 PMC153076

[ref22] Newberry K. J. Nakano S. Zuber P. Brennan R. G. (2005). Crystal structure of the *Bacillus subtilis* anti-alpha, global transcriptional regulator, Spx, in complex with the α C-terminal domain of RNA polymerase. Proc. Natl. Acad. Sci. U. S. A. 102, 15839–15844. doi: 10.1073/pnas.0506592102, PMID: 16249335 PMC1266077

[ref23] Nicolas P. Mäder U. Dervyn E. Rochat T. Leduc A. Pigeonneau N.. (2012). Condition-dependent transcriptome reveals high-level regulatory architecture in *Bacillus subtilis*. Science 335, 1103–1106. doi: 10.1126/science.1206848, PMID: 22383849

[ref24] Oda Y. Huang K. Cross F. R. Cowburn D. Chait B. T. (1999). Accurate quantitation of protein expression and site-specific phosphorylation. Proc. Natl. Acad. Sci. USA 96, 6591–6596. doi: 10.1073/pnas.96.12.6591, PMID: 10359756 PMC21959

[ref25] Pedersen T. L. (2024). *Patchwork: the composer of plots*. Available online at: https://CRAN.R-project.org/package=patchwork.

[ref26] Petersohn A. Bernhardt J. Gerth U. Höper D. Koburger T. Völker U.. (1999). Identification of ςB-dependent genes in *Bacillus subtilis* using a promoter consensus-directed search and oligonucleotide hybridization. J. Bacteriol. 181, 5718–5724. doi: 10.1128/jb.181.18.5718-5724.1999, PMID: 10482513 PMC94092

[ref27] R Core Team. (2024). *R: a language and environment for statistical computing. R foundation for statistical computing*; R Core Team. Available online at: https://www.R-project.org/.

[ref28] Reder A. Hentschker C. Steil L. Gesell Salazar M. Hammer E. Dhople V. M.. (2024). MassSpecPreppy—an end-to-end solution for automated protein concentration determination and flexible sample digestion for proteomics applications. Proteomics 24:e2300294. doi: 10.1002/pmic.202300294, PMID: 37772677

[ref29] Rhee H. W. Zou P. Udeshi N. D. Martell J. D. Mootha V. K. Carr S. A.. (2013). Proteomic mapping of mitochondria in living cells via spatially-restricted enzymatic tagging. Science 339, 1328–1331. doi: 10.1126/science.1230593, PMID: 23371551 PMC3916822

[ref30] Ritchie M. E. Phipson B. Wu D. Hu Y. Law C. W. Shi W.. (2015). Limma powers differential expression analyses for RNA-sequencing and microarray studies. Nucleic Acids Res. 43:e47. doi: 10.1093/nar/gkv007, PMID: 25605792 PMC4402510

[ref31] Roux K. J. Kim D. I. Raida M. Burke B. (2012). A promiscuous biotin ligase fusion protein identifies proximal and interacting proteins in mammalian cells. J. Cell Biol. 196, 801–810. doi: 10.1083/jcb.201112098, PMID: 22412018 PMC3308701

[ref32] Schmidt T. G. M. Batz L. Bonet L. Carl U. Holzapfel G. Kiem K.. (2013). Development of the twin-strep-tag® and its application for purification of recombinant proteins from cell culture supernatants. Protein Expr. Purif. 92, 54–61. doi: 10.1016/j.pep.2013.08.021, PMID: 24012791

[ref33] Senior A. W. Evans R. Jumper J. Kirkpatrick J. Sifre L. Green T.. (2020). Improved protein structure prediction using potentials from deep learning. Nature 577, 706–710. doi: 10.1038/s41586-019-1923-7, PMID: 31942072

[ref34] Shevchenko A. Wilm M. Vorm O. Mann M. (1996). Mass spectrometric sequencing of proteins from Silver-stained polyacrylamide gels. Anal. Chem. 68, 850–858. doi: 10.1021/ac950914h, PMID: 8779443

[ref35] Sievert C. (2020). Interactive web-based data visualization with R, Plotly, and shiny. Boca Raton, FL: Chapman and Hall, CRC.

[ref36] Sinz A. (2003). Chemical cross-linking and mass spectrometry for mapping three-dimensional structures of proteins and protein complexes. J. Mass Spectrom. 38, 1225–1237. doi: 10.1002/jms.559, PMID: 14696200

[ref37] Slowikowski K. (2024). *Ggrepel: automatically position non-overlapping text labels with “Ggplot2*. Available online at: https://CRAN.R-project.org/package=ggrepel.

[ref38] Smith G. P. (1985). Filamentous fusion phage: novel expression vectors that display cloned antigens on the virion surface. Science 228, 1315–1317. doi: 10.1126/science.4001944, PMID: 4001944

[ref39] Stryer L. Haugland R. P. (1967). Energy transfer: a spectroscopic ruler. Proc. Natl. Acad. Sci. U. S. A. 58, 719–726. doi: 10.1073/pnas.58.2.719, PMID: 5233469 PMC335693

[ref40] Sutherland B. W. Toews J. Kast J. (2008). Utility of formaldehyde cross-linking and mass spectrometry in the study of protein-protein interactions. J. Mass Spectrom. 43, 699–715. doi: 10.1002/jms.1415, PMID: 18438963

[ref41] Vega-Cabrera L. A. Guerrero A. Rodríguez-Mejía J. L. Tabche M. L. Wood C. D. Gutiérrez-Rios R. M.. (2017). Analysis of Spo0M function in *Bacillus subtilis*. PLoS One 12:e0172737. doi: 10.1371/journal.pone.0172737, PMID: 28234965 PMC5325327

[ref42] Wickham H. (2011). Testthat: get started with testing. R J. 3, 5–10. doi: 10.32614/RJ-2011-002

[ref43] Wickham H. Averick M. Bryan J. Chang W. McGowan L. François R.. (2019). Welcome to the tidyverse. J Open Source Softw. 4:1686. doi: 10.21105/joss.01686

[ref44] Wiseman T. Williston S. Brandts J. F. Lin L. N. (1989). Rapid measurement of binding constants and heats of binding using a new titration calorimeter. Anal. Biochem. 179, 131–137. doi: 10.1016/0003-2697(89)90213-3, PMID: 2757186

[ref45] Zuber P. (2004). Spx-RNA polymerase interaction and global transcriptional control during oxidative stress 186, 1911–1918. doi: 10.1128/jb.186.7.1911-1918.2004, PMID: 15028674 PMC374421

