# Supplementary material for: TIE-UP-SIN: a novel method for enhanced identification of protein–protein interactions
Source: Front Microbiol. 2025 Sep 1;16:1657647. doi: 10.3389/fmicb.2025.1657647 (PMC12434083; doi:10.3389/fmicb.2025.1657647)
Supplement: Supplementary file 1 [file Data_Sheet_1.pdf]

Supplemental Data

Supplemental S1-S3 and Supplemental Figures 1-5 are here in this pdf file. Protein databases and the R-script are included in the extra Supplemental files.

Supplemental Figure 1:

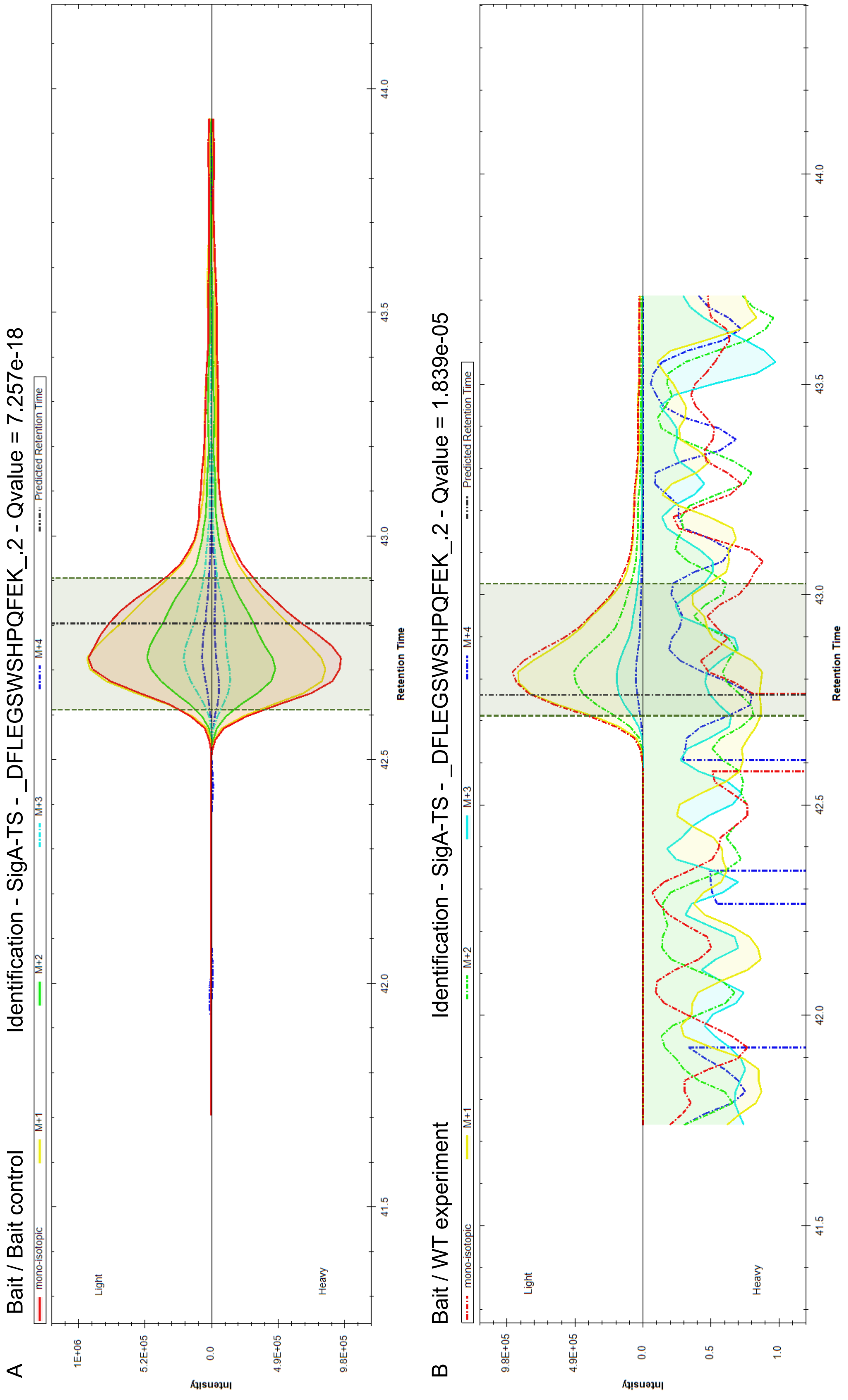

Supplemental Figure 1: MS1 XICs of one SigA-TS peptide's isotopoloques from a Bait/Bait control replicate (A) and from a Bait/WT experiment replicate (B).

Supplemental Figure 2

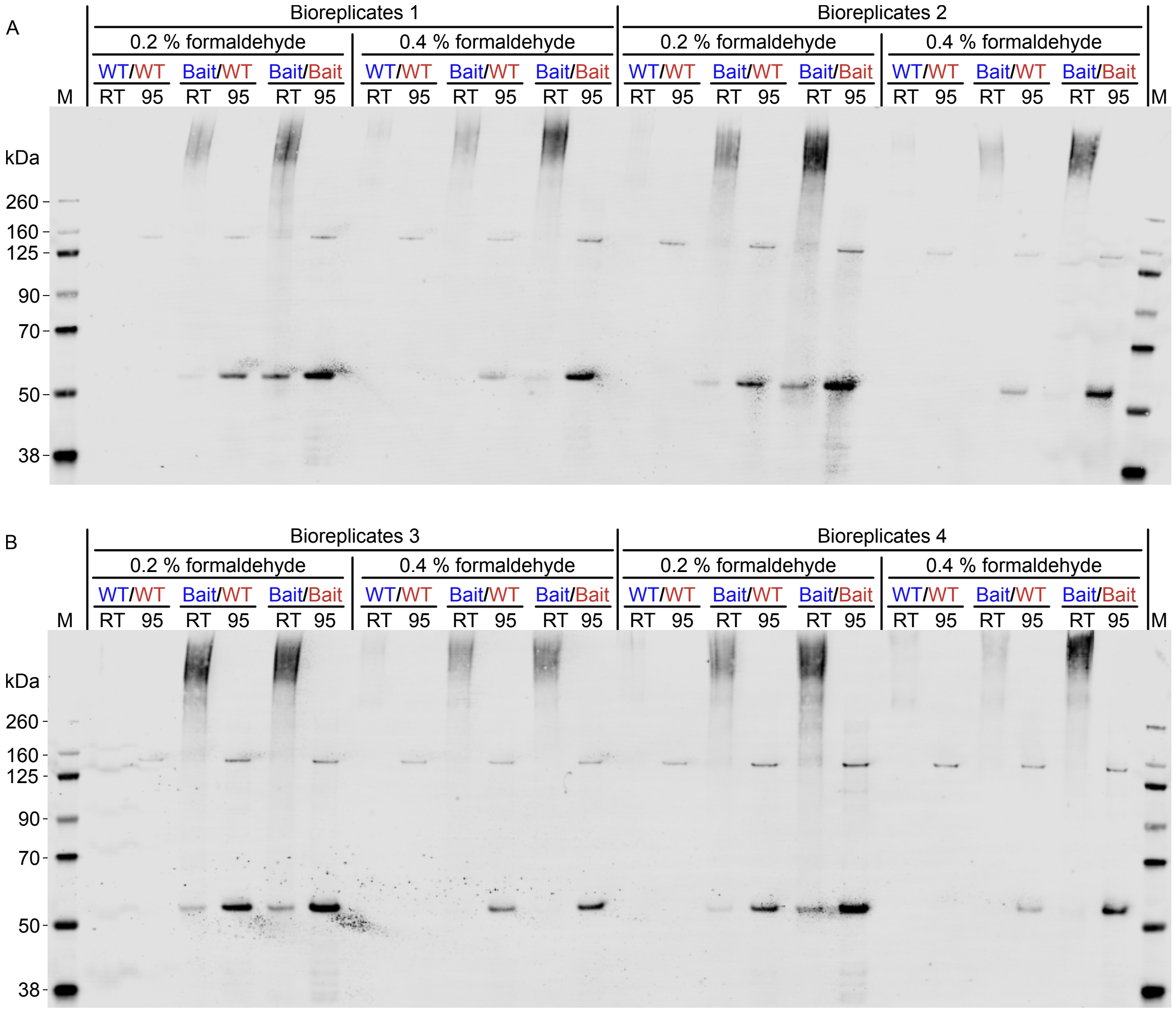

Supplemental Figure 2: Western blot analysis of all biological replicates of the control condition experiment. In (A) for biological replicates 1 + 2 and in (B) for biological replicates 3 + 4. Samples were cross-linked with either 0.2 % or 0.4 % (w/v) formaldehyde. Samples were created by mixing a N14 light labeled sample with an N15 heavy labeled sample in a 1:1 ratio. Eluates were kept at room temperature for 2 h (RT) or were kept at 95 °C for 2 h. SigA-TS is the signal at around 50 kDa and the signal at 130 kDa corresponds to PycA. 4 µl of each eluate were used for the SDS-PAGE. M: Chameleon® Duo Pre-stained Protein Ladder. The Twin-Strep tag was detected with the TactinXT CW800 NIR conjugate. Fluorescence was measured with the CLx Odyssey.

Supplemental Figure 3:

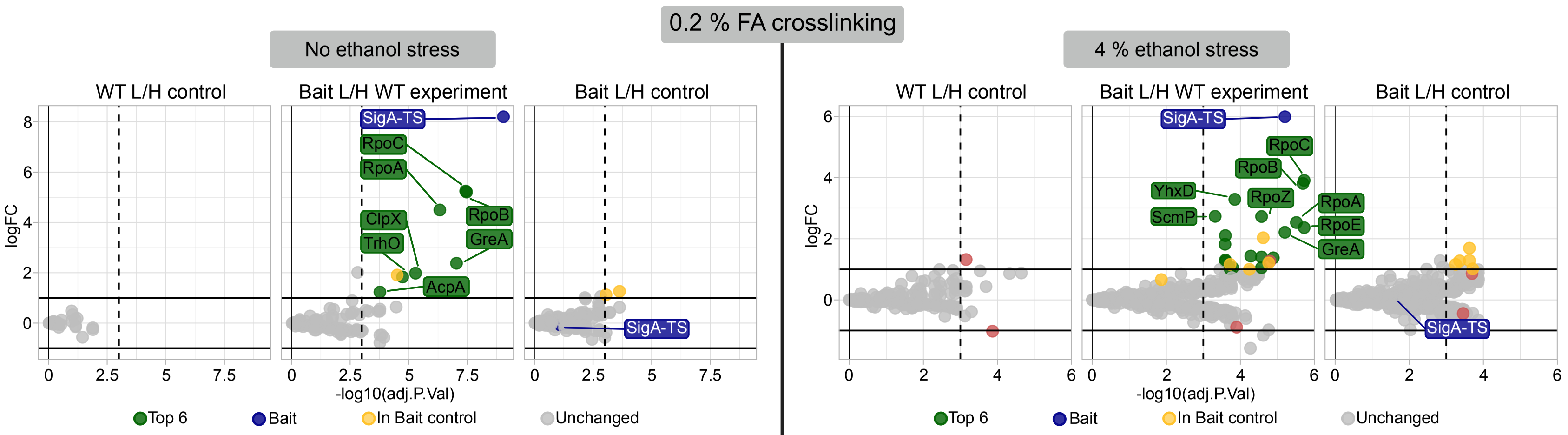

Protein Name	Protein Description	Status	Sample	Enrichment	Adjusted P.-Value
SigA_TS	Sigma-factor SigA with twinStrepTag	Bait	Bait L/H WT experiment	294.65	9.11E-10
RpoC	DNA-directed RNA polymerase subunit beta'	Top 7	Bait L/H WT experiment	38.02	3.71E-08
RpoB	DNA-directed RNA polymerase subunit beta	Top 7	Bait L/H WT experiment	37.00	3.42E-08
RpoA	DNA-directed RNA polymerase subunit alpha	Top 7	Bait L/H WT experiment	22.57	4.71E-07
GreA	Transcription elongation factor GreA	Top 7	Bait L/H WT experiment	5.20	9.19E-08
TrhO	tRNA uridine(34) hydroxylase	Top 7	Bait L/H WT experiment	3.95	5.11E-06
ClpX	ATP-dependent Clp protease ATP-binding subunit ClpX	Top 7	Bait L/H WT experiment	3.56	1.82E-05
AcpA	Acyl carrier protein	Top 7	Bait L/H WT experiment	2.35	1.69E-04
SigA_TS	Sigma-factor SigA with twinStrepTag	Bait	Bait L/H control	0.95	7.99E-02

Protein Name	Protein Description	Status	Sample	Enrichment	Adjusted P.-Value
SigA_TS	Sigma-factor SigA with twinStrepTag	Bait	Bait L/H WT experiment	63.57	6.26E-06
RpoC	DNA-directed RNA polymerase subunit beta'	Top 13	Bait L/H WT experiment	15.02	1.89E-06
RpoB	DNA-directed RNA polymerase subunit beta	Top 13	Bait L/H WT experiment	14.01	2.03E-06
YhxD	Uncharacterized oxidoreductase YhxD	Top 13	Bait L/H WT experiment	9.77	1.41E-04
ScmP	N-acetylcysteine deacetylase	Top 13	Bait L/H WT experiment	6.65	4.78E-04
RpoZ	DNA-directed RNA polymerase subunit omega	Top 13	Bait L/H WT experiment	6.63	2.67E-05
RpoA	DNA-directed RNA polymerase subunit alpha	Top 13	Bait L/H WT experiment	5.79	3.06E-06
RpoE	DNA-directed RNA polymerase subunit delta	Top 13	Bait L/H WT experiment	5.15	1.89E-06
GreA	Transcription elongation factor GreA	Top 13	Bait L/H WT experiment	4.63	6.26E-06
PyrF	Orotidine 5'-phosphate decarboxylase	Top 13	Bait L/H WT experiment	4.30	2.53E-04
IowL	scyllo-inositol 2-dehydrogenase (NADP(+)) IolW	Top 13	Bait L/H WT experiment	3.53	2.60E-04
Scp2	Stress response protein SCP2	Top 13	Bait L/H WT experiment	2.70	5.22E-05
FolD	Bifunctional protein FolD	Top 13	Bait L/H WT experiment	2.64	2.67E-05
YtxH	Uncharacterized protein YtxH	Top 13	Bait L/H WT experiment	2.60	1.29E-05
SigA_TS	Sigma-factor SigA with twinStrepTag	Bait	Bait L/H control	1.06	3.03E-02

Supplemental Figure 3: Volcano plots of the enrichment analysis from the samples crosslinked with 0.2 % FA. Filter parameter were as followed: WT_FC = 2; Bait_FC = 2; ion_Q = 0.05; CDC = 1; ion_CV = 0.3; NrPEP = 2; seqcov = 30; FC = 2; Pvalue = 0.001

Supplemental Figure 4:

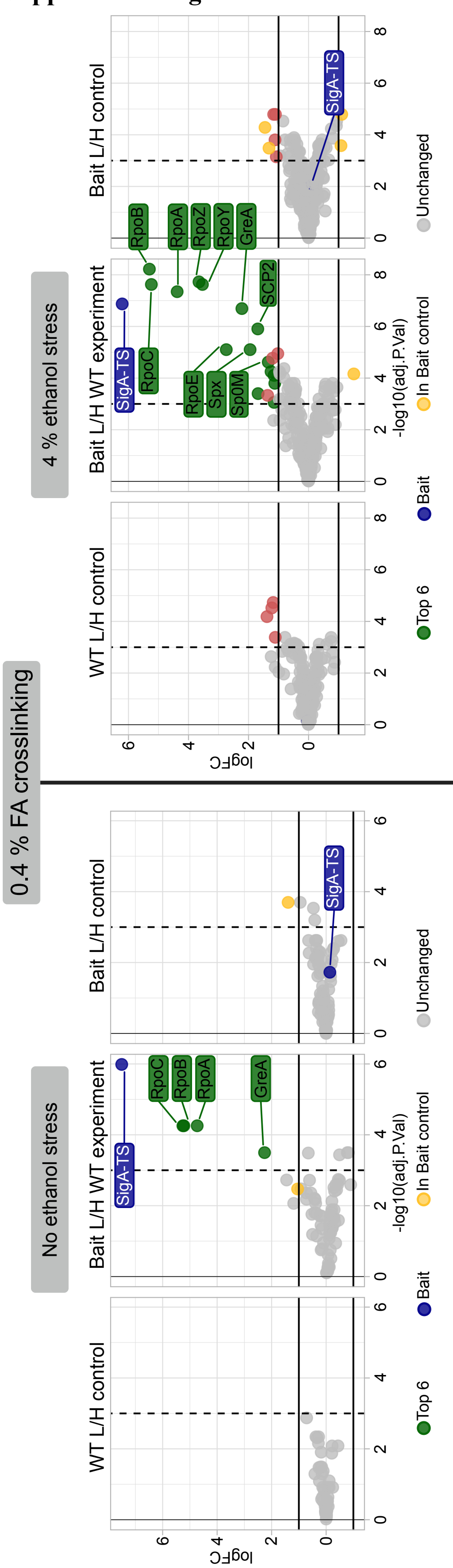

Protein Name	Protein Description	Status	Sample	Enrichment	Adjusted P-Value
SigA_TS	Sigma-factor SigA with twinStrepTag	Bait	Bait L/H WT experiment	181.26	1.03E-06
RpoC	DNA-directed RNA polymerase subunit beta'	Top 4	Bait L/H WT experiment	38.24	5.55E-05
RpoB	DNA-directed RNA polymerase subunit beta	Top 4	Bait L/H WT experiment	37.32	5.55E-05
RpoA	DNA-directed RNA polymerase subunit alpha	Top 4	Bait L/H WT experiment	26.57	5.55E-05
GreA	Transcription elongation factor GreA	Top 4	Bait L/H WT experiment	4.79	3.18E-04
SigA_TS	Sigma-factor SigA with twinStrepTag	Bait	Bait L/H control	0.91	1.88E-02

Protein Name	Protein Description	Status	Sample	Enrichment	Adjusted P-Value
SigA_TS	Sigma-factor SigA with twinStrepTag	Bait	Bait L/H WT experiment	74.03	1.34E-07
RpoB	DNA-directed RNA polymerase subunit beta	Top 11	Bait L/H WT experiment	39.48	6.03E-09
RpoC	DNA-directed RNA polymerase subunit beta'	Top 11	Bait L/H WT experiment	37.72	2.37E-08
RpoA	DNA-directed RNA polymerase subunit alpha	Top 11	Bait L/H WT experiment	20.79	4.50E-08
RpoZ	DNA-directed RNA polymerase subunit omega	Top 11	Bait L/H WT experiment	12.56	1.88E-08
RpoY	DNA-directed RNA polymerase subunit epsilon	Top 11	Bait L/H WT experiment	11.60	2.37E-08
RpoE	DNA-directed RNA polymerase subunit delta	Top 11	Bait L/H WT experiment	6.69	7.91E-06
GreA	Transcription elongation factor GreA	Top 11	Bait L/H WT experiment	4.66	2.03E-07
Spx	Global transcriptional regulator Spx	Top 11	Bait L/H WT experiment	3.86	7.91E-06
Scp2	Stress response protein SCP2	Top 11	Bait L/H WT experiment	3.23	1.25E-06
YwjC	Uncharacterized protein YwjC	Top 11	Bait L/H WT experiment	3.21	3.98E-04
SpOM	Sporulation-control protein spo0M	Top 11	Bait L/H WT experiment	2.54	2.40E-05
SigA_TS	Sigma-factor SigA with twinStrepTag	Bait	Bait L/H control	0.92	8.04E-03

Supplemental Figure 4: Volcano plots of the enrichment analysis from the samples crosslinked with 0.4 % FA. Filter parameter were as followed: WT_FC = 2; Bait_FC = 2; ion_Q = 0.05; CDC = 1; ion_CV = 0.3; NrPEP = 2; seqcov = 30; FC = 2; Pvalue = 0.001

Supplemental Figure 5:

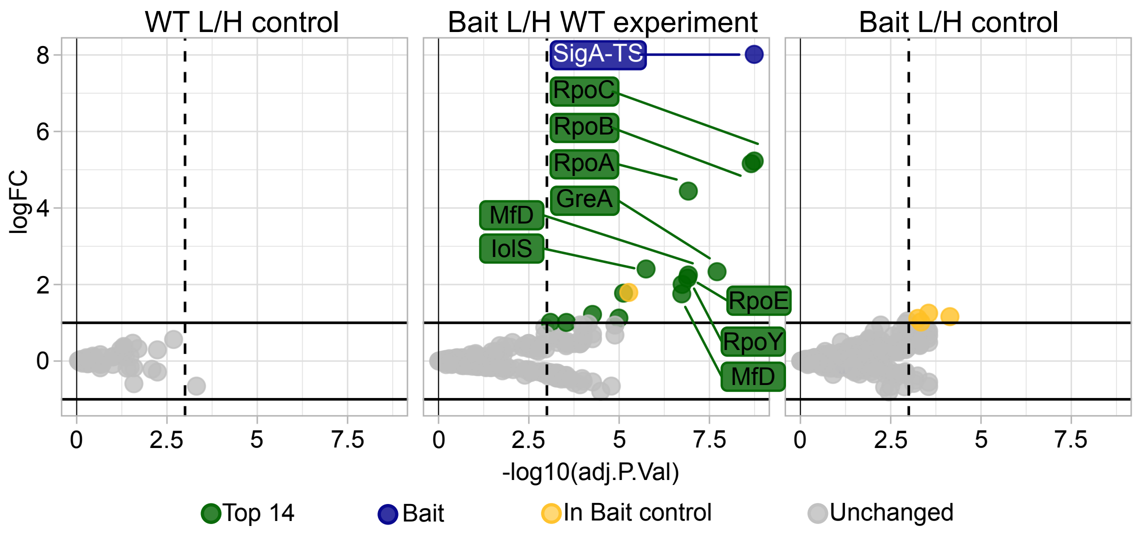

Protein Name	Protein Description	Status	Sample	Enrichment	Adjusted P.-Value
SigA_TS	Sigma-factor SigA with twinStrepTag	Bait	Bait L/H WT experiment	258.68	1.82E-09
RpoC	DNA-directed RNA polymerase subunit beta'	Top X	Bait L/H WT experiment	37.46	1.82E-09
RpoB	DNA-directed RNA polymerase subunit beta	Top X	Bait L/H WT experiment	35.74	2.21E-09
RpoA	DNA-directed RNA polymerase subunit alpha	Top X	Bait L/H WT experiment	21.73	1.21E-07
IolS	Aldo-keto reductase IolS	Top X	Bait L/H WT experiment	5.30	1.80E-06
GreA	Transcription elongation factor GreA	Top X	Bait L/H WT experiment	5.05	1.95E-08
MfD	Transcription-repair-coupling factor	Top X	Bait L/H WT experiment	4.75	1.21E-07
RpoE	DNA-directed RNA polymerase subunit delta	Top X	Bait L/H WT experiment	4.49	1.27E-07
RpoY	DNA-directed RNA polymerase subunit epsilon	Top X	Bait L/H WT experiment	4.01	1.80E-07
PurA	Adenylosuccinate synthetase	In Bait Ctrl	Bait L/H WT experiment	3.47	5.40E-06
ScP2	Stress response protein SCP2	Top X	Bait L/H WT experiment	3.42	7.55E-06
ClpX	ATP-dependent Clp protease ATP-binding subunit ClpX	Top X	Bait L/H WT experiment	3.38	1.84E-07
AcpA	Acyl carrier protein	Top X	Bait L/H WT experiment	2.33	5.46E-05
HiS8	Histidinol-phosphate aminotransferase	Top X	Bait L/H WT experiment	2.18	1.02E-05
HtpG	Chaperone protein HtpG	Top X	Bait L/H WT experiment	2.02	8.00E-04
SyfA	Phenylalanine--tRNA ligase alpha subunit	Top X	Bait L/H WT experiment	2.01	2.88E-04
SigA_TS	Sigma-factor SigA with twinStrepTag	Bait	Bait L/H control	0.94	6.47E-02

Supplemental Figure 5: TIE-UP-SIN results for SigA-TS under control conditions and crosslinked with 0.2 % (w/v) formaldehyde. For less stringent filtering the parameters ion_CV was increased to 0.3, the protein sequence coverage was decreased to 20 % and the remaining parameters stayed the same. Volcano plots depict the $\log_2$ enrichment of proteins in the WT L/H control (left), the Bait/WT experiment (middle), and the Bait L/H control (right). The x-axis shows the $\log_2$ fold enrichment, and the y-axis indicates statistical significance (-$\log_{10}$ adjusted P-Value). Grey dots represent detected but L/H ratio wise unchanged proteins, while colored markers highlight

significantly enriched proteins based on the adjusted p-value threshold (vertical dashed line) and the enrichment threshold (horizontal solid line). Highlighted proteins, such as SigA-TS (blue), RpoC, RpoB, RpoA, HelD, and GreA (green) met these thresholds and are labeled. Yellow and red dots indicate proteins that exceeded the thresholds but were enriched in one of the two control samples and thus were excluded from the list of potential interaction partners. Below the plots, a summary table details the names, descriptions, status, sample, and adjusted p-values of the highlighted proteins. Because of visual clarity reasons only the top 5 most enriched proteins are labeled in the plot. Information about the other Top X proteins is found in the corresponding table.

Supplementals S1-S3

S1 – SigA Construction

Table S1: Primer used for the SigA mutant construction

Primer Name	Sequence (5´- 3´)
ErmB_+SD_for	AGGAGGAGTTTAAAATGAACAAAAATATAAAATATTCTC
ErmB_just_rev	TTATTTCCTCCCGTTAAATAATAG
T_Strep_Ery_for	GGATCCTGGTCACATCCACAATTTGAAAAAGGTGGTGGTTCTGGTGGTGGTTCTGGTGGTTCAGCATGGTCACATCCGCAATTTGAAAAATAATGAGGAGGAGTTTAAAATGAACAAAAATATAAAATATTCTC
sigA_up_for	ATTCAGCAGCTTGCCAAAGC
sigA_up_rev	CAAATTGTGGATGTGACCAGGATCCTTCAAGGAAATCTTTCAAACGTTTAC
sigA_do_for	CTATTATTTAACGGGAGGAAATAAGATGGAACGGGTCTTGAAG
sigA_do_rev	CTCCATTCGGTATGTACTCC
sigA_seq_for	GTATTGCGTCTTCGATTCGG
sigA_seq_rev	CAGTTTCGTCGTTAAATGCC

S2 – MS settings

Supplementary Table S2: Detailed parameters for LC-MS/MS measurement.

***R**everse **P**hase **L**iquid **C**hromatography (RPLC)*	
Instrument	Ultimate 3000 RSLC (Thermo Scientific)
Trap column	75 µm inner diameter, packed with 3 µm C18 particles<br>(Acclaim PepMap100, Thermo Scientific)
Analytical column	Accucore 150-C18, (Thermo Fisher Scientific)<br>25 cm x 75 µm, 2,6 µm C18 particles, 150 Å pore size
Buffer system	Binary buffer system consisting of 0.1 % acetic acid in HPLC-grade water (solvent A) and 100 % ACN in 0.1 % acetic acid (solvent B)
Flow rate	300 nl/min
Gradient	Linear gradient of buffer B from 2 % up to 25 %<br>0 min-2 % B<br>2 min-5 % B<br>10 min-7 % B<br>40 min-25 % B<br>45 min-40 % B<br>47 min-90 % B<br>53 min-90 % B<br>55 min-2 % B<br>65 min-2 % B
Gradient duration (linear gradient)	30 min
Column oven temperature	40 °C

Mass Spectrometry	
Instrument	Orbitrap Exploris 480
Electrospray	Nanospray Flex™
Operation mode	Data-independent

MS scan resolution	120000
AGC target	3e6 (300 %)
Maximum ion injection time for the MS scan	60 ms
Scan range	350 to 1200 m/z
Microscans	1
Polarity	Positive
RF Lens	50 %
Spectra data type	Profile
Dia Properties (MS2)	
Resolution	30000
Maximum ion rejection time for the MS/MS scans	Auto
Normalized AGC traget	3e6
Spectra data type	Profile
Microscans	1
Isolation window	34
Isolation window width	25 m/z
Window overlay	2 m/z
Fixed first mass	200
HCD collision energy	30 %

S3 – MS settings

Supplementary Table S3 Detailed parameters for Spectronaut (version 17) search.

```
├─ DIA Analysis\Calibration
│  ├─ MZ Extraction Strategy:    Maximum Intensity
│  ├─ Allow source specific iRT Calibration: True
│  ├─ Precision iRT: True
│  │  ├─ Exclude De-amidated Peptides: True
│  │  └─ iRT <-> RT Regression Type:   Local (Non-Linear) Regression
│  ├─ MS1 Mass Tolerance Strategy:     System Default
│  └─ MS2 Mass Tolerance Strategy:     System Default
├─ DIA Analysis\Identification
│  ├─ Precursor Qvalue Cutoff:   0.001
│  ├─ Precursor PEP Cutoff:      0.2
│  ├─ Protein Qvalue Cutoff (Experiment):    0.01
│  ├─ Protein Qvalue Cutoff (Run):     0.05
│  ├─ Protein PEP Cutoff:  0.75
│  ├─ Single Hit Definition:     By Stripped Sequence
│  ├─ Exclude Single Hit Proteins:     False
│  ├─ Exclude Duplicate Assays:  True
│  ├─ Exclude Predicted Fragment Scores:     False
│  ├─ Generate Decoys:     True
│  │  ├─ Decoy Generation Method:      Mutated
│  │  │  └─ Preferred Fragment Source: NN Predicted Fragments
│  │  └─ Decoy Limit Strategy:   Dynamic
│  │     └─ Library Size Fraction:     0.1
│  └─ Pvalue Estimator:    Kernel Density Estimator
├─ DIA Analysis\Pipeline Mode
│  ├─ Generate SNE File:   True
│  │  └─ Store Ion traces in SNE:      True
│  ├─ Post Analysis Reports:
│  │  ├─ CV Density Line Chart:  True
│  │  ├─ CVs Below X Bar Chart:  True
│  │  ├─ Data Completeness Bar Chart:  True
│  │  ├─ Run Identifications Bar Chart:      True
│  │  └─ Scoring Histograms:     True
│  ├─ Report Schema: C_FunGene_complex (Normal)
│  └─ Reporting Unit:      Across Experiment
├─ DIA Analysis\Post Analysis
│  ├─ Differential Abundance Testing:  Paired t-test
│  │  ├─ Group-Wise Testing Correction:      False
│  │  ├─ Log2 Ratio Candidate Filter:  0.58
│  │  └─ Confidence Candidate Filter:  Qvalue
│  │     └─ Confidence:    0.05
│  ├─ Differential Abundance Grouping: Major Group (Quantification
Settings)
│  │  └─ Smallest Quantitative Unit:   Precursor Ion (Quantification
Settings)
│  │     └─ Use All MS-Level Quantities:     False
│  ├─ Calculate Explained TIC:   None
│  ├─ Calculate Sample Correlation Matrix:   True
│  └─ Hierarchical Clustering:   True
│     ├─ Distance Metric:  Manhattan Distance
│     ├─ Linkage Strategy: Ward's Method
│     ├─ Order Runs by Clustering:     True
│     └─ Z-score Transformation: False
├─ DIA Analysis\Protein Inference
```

```
│  └─ Protein Inference Workflow:      Automatic
│     └─ Inference Algorithm:    IDPicker
├─ DIA Analysis\PTM Workflow
│  └─ PTM Localization:    True
│     ├─ Probability Cutoff:     0.75
│     └─ PTM Analysis:     True
│        ├─ Hierarchical Clustering:   False
│        ├─ Multiplicity:  True
│        ├─ Flanking Region:     7
│        └─ PTM Consolidation:   Sum
├─ DIA Analysis\Quantification
│  ├─ Precursor Filtering: Identified (Qvalue)
│  │  └─ Imputation Strategy:    Use Background Signal
│  ├─ Proteotypicity Filter:     None
│  ├─ Protein LFQ Method:  Automatic
│  ├─ Quantity MS Level:   MS2
│  ├─ Quantity Type: Area
│  ├─ Cross-Run Normalization:   False
│  ├─ Quantification window:     Not Synchronized (SN 17)
│  ├─ Interference Correction:   True
│  │  ├─ Only Identified Peptides:     True
│  │  ├─ Exclude All Multi-Channel Interferences:  True
│  │  ├─ MS1 Min:    2
│  │  └─ MS2 Min:    3
│  ├─ Major (Protein) Grouping:  by Protein Group Id
│  ├─ Minor (Peptide) Grouping:  by Stripped Sequence
│  ├─ Major Group Quantity:      Mean peptide quantity
│  ├─ Major Group Top N:   False
│  ├─ Minor Group Quantity:      Sum precursor quantity
│  └─ Minor Group Top N:   False
├─ DIA Analysis\Workflow
│  ├─ Method Evaluation:   False
│  ├─ MS2 DeMultiplexing:  Automatic
│  ├─ Profiling Strategy:  iRT Profiling
│  │  ├─ Carry-over exact Peak Boundaries:   False
│  │  ├─ Profiling Row Selection:      Minimum Qvalue Row Selection
│  │  │  └─ Qvalue Threshold:    0.001
│  │  └─ Profiling Target Selection:   Profile only non-identified
Precursors
│  │     ├─ Identification Criterion:  Qvalue
│  │     └─ Threshold:     0.001
│  ├─ Run Limit for directDIA Library: -1
│  └─ Unify Peptide Peaks Strategy:    Select corresponding Peak
├─ DIA Analysis\XIC Extraction
│  ├─ XIC IM Extraction Window:  Dynamic
│  │  └─ Correction Factor:      1
│  ├─ XIC RT Extraction Window:  Dynamic
│  │  └─ Correction Factor:      1
│  ├─ MS1 Mass Tolerance Strategy:     Dynamic
│  │  └─ Correction Factor:      1
│  └─ MS2 Mass Tolerance Strategy:     Dynamic
│     └─ Correction Factor:      1
├─ Pulsar Search\Identification
│  ├─ PSM FDR: 0.01
│  ├─ Peptide FDR:   0.01
│  ├─ Protein Group FDR:   0.01
│  ├─ directDIA Workflow:  directDIA+ (Deep)
│  └─ PTM Localization Filter:   False
├─ Pulsar Search\Labeling
│  └─ Channels:
│     ├─ Channel 1:  True
```

```
│       │  └─ Labels in This Channel::
│       ├─ Channel 2:  True
│       │  └─ Labels in This Channel::   Label:15N(1), Label:15N(2),
Label:15N(3), Label:15N(4)
│       └─ Channel 3:  False
├─ Pulsar Search\Modifications
│  ├─ Max Variable Modifications:      5
│  └─ Select Modifications:
│     ├─ Fixed Modifications::
│     └─ Variable Modifications: :     Oxidation (M)
├─ Pulsar Search\Peptides
│  ├─ Enzymes / Cleavage Rules:  Trypsin/P
│  ├─ Digest Type:   Specific
│  ├─ Max Peptide Length:  52
│  ├─ Min Peptide Length:  7
│  ├─ Missed Cleavages:    2
│  └─ Toggle N-terminal M: True
├─ Pulsar Search\Result Filters
│  ├─ Fragment Ions:
│  │  ├─ Ion AA Length:    True
│  │  │  └─ N: 3
│  │  ├─ Ion Charge: False
│  │  ├─ Ion Loss Type:    False
│  │  ├─ Ion Type:   False
│  │  ├─ m/z : True
│  │  │  ├─ Max:     1800
│  │  │  └─ Min:     300
│  │  └─ Relative Intensity:     True
│  │     └─ Min:     5
│  └─ Precursors:
│     ├─ Amino Acids:      False
│     ├─ Best N Fragments per Peptide: True
│     │  ├─ Max:     10
│     │  └─ Min:     6
│     ├─ Best N Peptides per Protein Group:  False
│     ├─ Channel Count:    False
│     ├─ FASTA Matched:    False
│     ├─ Missed Cleavage:  False
│     ├─ Modifications:    None
│     ├─ Peptide Charge:   False
│     └─ Proteotypicity:   False
├─ Pulsar Search\Speed-Up
│  ├─ IM DFD Processing:
│  │  └─ Use Dynamic IM Peak Filter:   True
│  │     └─ Target TIC Fraction: 0.9
│  └─ MS2 Index:     Automatic
├─ Pulsar Search\Tolerances
│  └─ Tolerance Parameters:
│     ├─ Thermo IonTrap:
│     │  ├─ Calibration Search:  Dynamic
│     │  │  ├─ MS1 Correction Factor:  1
│     │  │  └─ MS2 Correction Factor:  1
│     │  └─ Main Search:   Dynamic
│     │     ├─ MS1 Correction Factor:  1
│     │     └─ MS2 Correction Factor:  1
│     ├─ Thermo Orbitrap:
│     │  ├─ Calibration Search:  Dynamic
│     │  │  ├─ MS1 Correction Factor:  1
│     │  │  └─ MS2 Correction Factor:  1
│     │  └─ Main Search:   Dynamic
│     │     ├─ MS1 Correction Factor:  1
```

```
│       │     └─ MS2 Correction Factor:  1
│       └─ TOF:
│           ├─ Calibration Search:  Dynamic
│           │  ├─ MS1 Correction Factor:  1
│           │  └─ MS2 Correction Factor:  1
│           └─ Main Search:   Dynamic
│              ├─ MS1 Correction Factor:  1
│              └─ MS2 Correction Factor:  1
└─ Pulsar Search\Workflow
   ├─ Fragment Ion Selection Strategy: Intensity Based
   ├─ In-Silico Generate Missing Channels:   True
   │  └─ Workflow:   label
   └─ Use DNN Predicted Ion Mobility:  Auto
```
